# CHIIMP: An automated high‐throughput microsatellite genotyping platform reveals greater allelic diversity in wild chimpanzees

**DOI:** 10.1002/ece3.4302

**Published:** 2018-07-16

**Authors:** Hannah J. Barbian, Andrew Jesse Connell, Alexa N. Avitto, Ronnie M. Russell, Andrew G. Smith, Madhurima S. Gundlapally, Alexander L. Shazad, Yingying Li, Frederic Bibollet‐Ruche, Emily E. Wroblewski, Deus Mjungu, Elizabeth V. Lonsdorf, Fiona A. Stewart, Alexander K. Piel, Anne E. Pusey, Paul M. Sharp, Beatrice H. Hahn

**Affiliations:** ^1^ Departments of Microbiology and Medicine Perelman School of Medicine University of Pennsylvania Philadelphia Pennsylvania; ^2^ Department of Anthropology Washington University in St. Louis St. Louis Missouri; ^3^ Gombe Stream Research Center Kigoma Tanzania; ^4^ Department of Psychology Franklin and Marshall College Lancaster Pennsylvania; ^5^ School of Natural Sciences and Psychology Liverpool John Moores University Liverpool UK; ^6^ Department of Evolutionary Anthropology Duke University Durham North Carolina; ^7^ Institute of Evolutionary Biology and Centre for Immunity Infection and Evolution University of Edinburgh Edinburgh UK

**Keywords:** high‐throughput STR genotyping, length homoplasy, *Pan troglodytes*, parentage analysis, short tandem repeats

## Abstract

Short tandem repeats (STRs), also known as microsatellites, are commonly used to noninvasively genotype wild‐living endangered species, including African apes. Until recently, capillary electrophoresis has been the method of choice to determine the length of polymorphic STR loci. However, this technique is labor intensive, difficult to compare across platforms, and notoriously imprecise. Here we developed a MiSeq‐based approach and tested its performance using previously genotyped fecal samples from long‐term studied chimpanzees in Gombe National Park, Tanzania. Using data from eight microsatellite loci as a reference, we designed a bioinformatics platform that converts raw MiSeq reads into locus‐specific files and automatically calls alleles after filtering stutter sequences and other PCR artifacts. Applying this method to the entire Gombe population, we confirmed previously reported genotypes, but also identified 31 new alleles that had been missed due to sequence differences and size homoplasy. The new genotypes, which increased the allelic diversity and heterozygosity in Gombe by 61% and 8%, respectively, were validated by replicate amplification and pedigree analyses. This demonstrated inheritance and resolved one case of an ambiguous paternity. Using both singleplex and multiplex locus amplification, we also genotyped fecal samples from chimpanzees in the Greater Mahale Ecosystem in Tanzania, demonstrating the utility of the MiSeq‐based approach for genotyping nonhabituated populations and performing comparative analyses across field sites. The new automated high‐throughput analysis platform (available at https://github.com/ShawHahnLab/chiimp) will allow biologists to more accurately and effectively determine wildlife population size and structure, and thus obtain information critical for conservation efforts.

## 1 INTRODUCTION

Microsatellites comprise short tandem repeats (STRs) of one to six base pairs, which are commonly used to profile DNA for a variety of applications ranging from cancer diagnosis to forensics (Bennett, 2000; Ellegren, 2004; Guichoux et al., 2011; Lynch & de la Chapelle, 2003). STR loci have a high mutation rate and vary in the number of their repeat motifs, due to slippage of the polymerase during DNA synthesis (Kelkar et al., 2010; Levinson & Gutman, 1987). Because of their ubiquity, high allelic diversity, and codominant inheritance, microsatellites are commonly used for individual identification, parentage analyses and population genetics (Balloux & Lugon‐Moulin, 2002; Jarne & Lagoda, 1996; Queller, Strassmann, & Hughes, 1993; Selkoe & Toonen, 2006). STR analysis can also be performed on samples containing little host DNA, such as hair and fecal samples, and has thus been the method of choice to genotype endangered primate species, which are typically sampled noninvasively (Constable, Ashley, Goodall, & Pusey, 2001; Constable, Packer, Collins, & Pusey, 1995; Morin, Wallis, Moore, Chakraborty, & Woodruff, 1993; Taberlet et al., 1997). An accurate determination of the number, distribution, and population connectivity of wild primates is essential for designing effective conservation measures to protect these species under increasing anthropogenic threat from habitat loss, disease, and hunting (Arandjelovic & Vigilant, 2018). However, census and population genetics studies of wild apes have been impeded by difficulties of accurately and cost‐effectively genotyping large numbers of noninvasively collected samples.

Until recently, the length of polymorphic STR loci has been determined by capillary electrophoresis, which compares the mobility of fluorescently labeled PCR products to a size standard of control fragments and thus yields only approximate results (e.g., a locus size of “167.5 bp”). Manual correction of such ambiguities can lead to arbitrary allele binning and inconsistent calls between experiments and/or investigators (Ewen et al., 2000; Weeks, Conley, Ferrell, Mah, & Gorin, 2002). In addition, amplification of STR loci frequently generates PCR artifacts, which are difficult to identify on electropherograms. These include stutter peaks, which are usually one repeat shorter than the correct STR allele and derive from Taq polymerase slippage (Hauge & Litt, 1993; Shinde, Lai, Sun, & Arnheim, 2003), split peaks which are caused by inconsistent A‐overhang addition (Schuelke, 2000), and artifactual peaks, which are the product of off‐target amplification and/or unspecific fluorescent signaling (Ewen et al., 2000; Fernando, Evans, Morales, & Melnick, 2001; Guichoux et al., 2011). Existing peak calling software often fails to differentiate erroneous from real peaks and frequently omits peaks of low height. Automatically called peaks must therefore be corrected manually, which is labor intensive and time‐consuming (Guichoux et al., 2011). Finally, multiplexing is restricted to only a few fluorescent labels, thus limiting the number of loci that can be analyzed simultaneously. As a consequence, capillary electrophoresis‐based STR genotyping is laborious, notoriously imprecise, and generally not useful for large sample sets or data sharing between different platforms and/or field sites (Pasqualotto, Denning, & Anderson, 2007).

To improve the accuracy and throughput of STR genotyping, investigators have begun to use next‐generation sequencing technologies to characterize amplified microsatellite loci. This approach is superior to capillary electrophoresis, as it yields unambiguous allele lengths regardless of protocol or sequencing platform. In addition, genotyping‐by‐sequencing (GBS) distinguishes alleles of the same size that contain substitutions or differ in length by a single nucleotide (Adams, Brown, & Hamilton, 2004). Although initially developed for human forensics (Fordyce et al., 2011; Van Neste, Van Nieuwerburgh, Van Hoofstat, & Deforce, 2012), GBS technologies have recently been used to genotype wild animals, including Atlantic cod (Vartia et al., 2016), brown bear (De Barba et al., 2017), boarfish (Farrell, Carlsson, & Carlsson, 2016), and muskrat (Darby, Erickson, Hervey, & Ellis‐Felege, 2016). These studies demonstrated the utility of GBS for molecular ecology applications (Darby et al., 2016; Farrell et al., 2016) and showed that even samples containing small quantities of host DNA, such as dung and hair, can be used for these analyses (De Barba et al., 2017). However, alleles were primarily called manually by visual inspection of read length histograms (Darby et al., 2016; Farrell et al., 2016; Vartia et al., 2016), and none of these studies have compared the performance of capillary electrophoresis and high‐throughput sequencing side‐by‐side to validate and improve the genotyping approach.

For nearly two decades, our group has been studying chimpanzees in Gombe National Park (Tanzania) to assess the long‐term impact of simian immunodeficiency virus (SIVcpz) infection on this wild‐living population (Keele et al., 2009; Rudicell et al., 2010; Santiago et al., 2003). To identify SIVcpz infected individuals, we developed noninvasive diagnostic assays that detect virus‐specific antibodies and nucleic acids by analysis of fecal samples. To reliably monitor the spread of SIVcpz in all three Gombe communities, we verified the individual origin of each fecal sample by microsatellite analysis at eight polymorphic STR loci. Thus, most Gombe chimpanzees have been repeatedly genotyped, resulting in a consensus genotype that has been used for paternity and kinship determinations, immunogenetics, microbiome analyses and behavioral studies (Barbian et al., 2018; Keele et al., 2009; Moeller et al., 2016; Rudicell et al., 2010; Santiago et al., 2003; Walker et al., 2017; Wroblewski et al., 2015).

Here, we used these multiply confirmed reference microsatellites as a guide to develop and iteratively improve a MiSeq‐based STR genotyping approach. To permit the direct comparison with previous capillary electrophoresis results, we determined the length of STR loci by sequencing PCR amplicons in their entirety, including both forward and reverse primers. We also developed a *Computational High‐throughput Individual Identification through Microsatellite Profiling* (CHIIMP) pipeline that detects and filters erroneous alleles and automatically generates a number of downstream analyses, such as allele length histograms, alignments of allele sequences, contamination heatmaps and genotype comparisons. By directly comparing the new CHIIMP‐derived genotypes to previously determined capillary electrophoresis results, we show that the new analysis tools, which are not included in any of the previously published STR genotyping pipelines, greatly improve the speed, cost, and accuracy of allele determinations.

## 2 MATERIALS AND METHODS

### 2.1 Chimpanzee fecal samples

Fecal samples were collected from wild‐living chimpanzees in Gombe National Park, including members of the Mitumba, Kasekela and Kalande communities, as well as the Greater Mahale Ecosystem (GME) in Tanzania as previously described (Keele et al., 2009; Rudicell et al., 2010, 2011; Santiago et al., 2003). Habituated Gombe chimpanzees have been under direct observation since the 1960s (Pusey, Pintea, Wilson, Kamenya, & Goodall, 2007; van Lawick‐Goodall, 1968), with prospective fecal sampling and SIVcpz diagnostics initiated in 1999 (Keele et al., 2009; Rudicell et al., 2010). Long‐term monitoring of nonhabituated chimpanzees in the GME began in 2008, with noninvasive SIVcpz screening implemented in 2009 (Rudicell et al., 2011). Gombe and GME fecal samples were collected 1:1 (vol/vol) in RNA*later* (Ambion), a high salt solution that preserves nucleic acids and allows storage and transport at room temperature. For individual identification, samples were routinely subjected to mitochondrial, sex, and microsatellite analyses, with up to eight STR loci characterized by capillary electrophoresis as described previously (Keele et al., 2009; Rudicell et al., 2010, 2011). All fieldwork has been approved by the Tanzania National Parks, the Tanzania Commission for Science and Technology, the Tanzania Wildlife Research Institute, and has followed the American Society of Primatologists’ Principles for Ethical Treatment of Nonhuman Primates.

### 2.2 Quantification of chimpanzee DNA

Fecal DNA was extracted from 0.5 ml of homogenized fecal suspension using the QIAamp DNA Stool Kit and the automated QIAcube system (Qiagen). Purified DNA was eluted in 200 μl water and stored at −20°C. Chimpanzee genomic DNA content was determined using a previously described *c‐myc* gene‐based quantitative (q)PCR (Morin, Chambers, Boesch, & Vigilant, 2001). Briefly, 2 μl DNA extract was added to 1× High Fidelity PCR Buffer, 3.5 mM MgSO_4_, 0.3 μM forward (5′‐GCCAGAGGAGGAACGAGCT‐3′) and reverse (5′‐GGGCCTTTTCATTGTTTTCCA‐3′) qPCR primers, 0.2 μM of a FAM‐labeled probe (FAM‐TGCCCTGCGTGACCAGATCC‐BHQ1), 0.2 mM dNTPs, 1× ROX Reference Dye, and 0.5 U Platinum Taq DNA Polymerase High Fidelity (Invitrogen). Each sample was run in triplicate on a 7900HT Fast Real‐Time PCR System, together with human genomic DNA standards of known concentration (the sequence of the particular *c‐myc* amplicon is identical between humans and chimpanzees). Negative “no‐template” controls were included in each run. Sequence Detection Systems version 2.3 software (Applied Biosystems) was used to quantify the host DNA content of each sample. As host DNA concentrations differed, approximately half of the samples were extracted on more than one occasion to generate enough material for all analyses.

### 2.3 Amplification of STR loci

Previous genotyping studies of Gombe and GME chimpanzees utilized eight STR loci containing tetranucleotide repeats (Constable et al., 2001; Keele et al., 2009; Rudicell et al., 2011). These included D18s536 (also termed locus A), D4s243 (locus B), D10s676 (locus C), D9s922 (locus D), D2s1326 (locus 1) D2s1333 (locus 2), D4s1627 (locus 3), and D9s905 (locus 4) (Supporting Information Table S1). To facilitate MiSeq sequencing of the amplified loci, we added MiSeq‐specific adapters to the 5′ end of both the forward (5′‐TCGTCGGCAGCGTCAGATGTGTATAAGAGACAG‐3′) and the reverse primer (5′‐GTCTCGTGGGCTCGGAGATGTGTATAAGAGACAG‐3′), respectively. Individual STR loci were amplified using 3–5 μl fecal DNA extract, 2.5 μl 10× AmpliTaq Gold Buffer, 1.75 μl 25 mM MgCl_2_, 1.5 μl 10 mM dNTPs, 0.5 μl 50 μg/ml BSA, 1.5 μl of 10 mM forward and reverse primers, and 0.25 μl AmpliTaq Gold polymerase (5 U/ml; Applied Biosystems) in a 25 μl reaction volume. Thermocycling was performed using an initial denaturation for 10 min at 94°C, followed by 50 cycles of 30 s at 94°C, 30 s at 54°C, and 45 s at 72°C, followed by a final extension of 10 min at 72°C.

Testing the sensitivity of MiSeq derived allele detection, we found that individual PCR reactions often produced only partial genotypes, while the combination of multiple amplicons from the same DNA sample generally yielded a more complete set of alleles. Consistent with previous studies (Morin et al., 2001), we also found that PCR amplification of less than 25 pg of host DNA generally failed to amplify STR loci. For all genotyping analyses, we thus included only DNA samples that contained more than 25 pg of chimpanzee DNA, amplified each STR locus on three independent occasions, and combined equal volumes of these replicate PCR reactions prior to MiSeq sequencing.

The eight STR loci were also amplified in one‐step and two‐step multiplex reactions. To minimize primer‐primer interactions, locus A, B, C and 3 primers were combined at an even ratio in one pool, while locus D, 1, 2, and 4 primers were similarly combined in a second pool. Fecal DNA was then amplified in two (rather than eight) different reactions, using the identical cycling conditions as for singleplex PCR. For two‐step multiplexing, 2 μl of a 1:100 dilution of the one‐step product was used as a template for a second round of PCR in which each locus was amplified individually using the same thermocycling conditions (Arandjelovic et al., 2009).

### 2.4 Library preparation and MiSeq sequencing

Following STR locus amplification, PCR products (individual or pooled) were diluted in nuclease‐free sterile water (1:10) and subjected to two rounds of PCR to add Illumina barcodes and enrich for properly indexed DNA products as described (Iyer et al., 2017). The resulting libraries were pooled, purified with Ampure Beads (Beckman Coulter), quantified using a Qubit Fluorometer (Thermo Scientific) and TapeStation 2200 (Agilent), and diluted to a final DNA concentration of 4 nM (Iyer et al., 2017). A randomly fragmented (adapter ligated) control library of PhiX DNA (Illumina) was added to increase read length diversity to ensure cluster recognition on the flow‐cell. Both PhiX control and STR amplicon libraries were adjusted to a final DNA concentration of 12 pM and mixed 1:1 prior to loading onto the sequencing reagent cartridge. All STR amplicons were sequenced in one direction using v2 chemistry (500 cycle kits) without fragmentation. This increased the length of the STR loci that could be analyzed to ~400 bp (instead of 2 × 250 paired‐end reads). Although 500 cycles are the theoretical maximum of the sequencing kit, we observed diminishing data quality between 350 and 400 cycles. We thus selected 375 forward and 51 reverse read cycles, using only the forward reads for analysis to preclude alignment artifacts of pairing reads in the repeat regions (the reverse reads were only used for MiSeq quality control). To maximize the number of amplicons sequenced per run, we used dual index multiplexing of samples.

### 2.5 CHIIMP analysis pipeline

Following MiSeq sequencing, read files were processed using standard methods. First, sample demultiplexing and FASTQ file generation was performed using the Illumina MiSeq Reporter software with default settings. Next, MiSeq adapter sequences were trimmed using cutadapt (Martin, 2011). The adapter trimmed forward reads from each read pair, which covered the entire STR amplicon, were then imported into the R package, which was used for all downstream analyses.

The CHIIMP analysis pipeline generates multi‐locus genotypes in three stages. First, each MiSeq sequence file is processed into unique sequences with relevant attributes, such as read counts, sequence length, and whether the sequence matches the locus‐specific forward primer, repeat motifs, and length range. Sequences are also queried for potential PCR artifacts, such as single nucleotide substitutions, indels, and stutter sequences introduced by Taq polymerase and sequencing errors. These artifacts are identified as comprising less than one‐third of the read counts of the corresponding allele. The 33% threshold was selected because inspection of known heterozygous loci revealed that all of the true second most frequent alleles contained more than that proportion of reads. Finally, for each sample and locus, the proportion of sequence reads of the total read count is determined. At this stage, data are kept for all loci to ensure flexible downstream processing, such as detecting cross‐locus contamination.

The second stage removes all sequences that do not match the locus attributes, such as the forward primer, repeat motif, and locus length, and/or contain likely PCR artifacts. In addition, only sequences comprising a minimum fraction of the total number of filtered reads (5%) are retained, and only loci with a total filtered read count above a customizable per‐sample read threshold (>500) are genotyped. Application of these filters determines the sample zygosity; if only one sequence passes these filters, the locus is reported as homozygous. However, if two or more sequences pass the filters, the two most abundant are kept and the sample is reported as heterozygous. The output at this stage includes a spreadsheet with the sequence content, read counts, sequence lengths, as well as other relevant information such as whether the sequence contains the correct repeat motif or was identified as a likely stutter sequence or other PCR artifact. Of note, all filters and thresholds are customizable, with the above parameters representing the default.

In the final stage, genotypes are assembled for all samples and loci, with quality control tables generated as output files (Supporting Information Figure S1). First, a summary genotype table is generated that lists sample designations for each row, STR loci for each column, and unique allele identifiers for each cell (Supporting Information Figure S1a). If specific allele codes are provided, the summary table will include these designations. If an allele does not match previous identifiers, the software will create a short name based on sequence length and content to identify these new alleles (e.g., sample 4781, locus C, allele 2 in Supporting Information Figure S1a). The similarity of genotypes is also depicted in a heatmap (Supporting Information Figure S1b), which groups closely related genotypes (Peakall & Smouse, 2006). In cases where genotypes of individuals are known, the program links samples with the corresponding individuals (Supporting Information Figure S1c). A heatmap shows the extent of similarity of every sample with every known genotype, thus allowing simple individual identification (Supporting Information Figure S1d). The program also generates a set of tables that flag alleles that require additional attention, such as loci where the stutter filter has been invoked, where more than two sequences passed the filter, where a large proportion of sequences was not contained in the identified alleles, and where homozygosity may reflect allelic dropout (Supporting Information Figure S1e). For each locus, the program creates a FASTA file of all allele sequences and an image of their alignment (Supporting Information Figure S1f) generated by the Bioconductor's MSA package (Bodenhofer, Bonatesta, Horejš‐Kainrath, & Hochreiter, 2015). In addition, a heatmap of sequence counts that match the locus‐specific forward primer for all samples and loci is generated (Supporting Information Figure S1g). For singleplex samples, this identifies sequences that match other loci and thus highlights potential cross‐locus contamination. For multiplexed samples, this shows the read distribution across different loci. Finally, histograms that show sequence length‐frequency distributions are saved as image files (Supporting Information Figure S1h). A summary file is created that combines all key results (sequences, read counts, etc.) for alleles for all samples and loci. This data output file is suitable for further analysis in R.

The new analysis platform, termed *Computational High‐throughput Individual Identification through Microsatellite Profiling* or CHIIMP, has been designed to allow customization of the number and sequence content of microsatellite loci to be analyzed. Particular locus attributes such as the expected locus length range, primer sequences, and repeat motif sequence can be specified in a simple text file. Thus, the software can be readily adapted to additional microsatellite loci, as long as the respective amplicons fall within the length limits of the particular sequence chemistry used. The software is also suitable to analyze multiplexed samples, which contain reads from several loci but are processed separately, again using the forward primers to select locus‐specific reads. No additional software is required other than providing a list of samples and loci prior to analysis. CHIIMP is available at https://github.com/ShawHahnLab/chiimp and can be installed on any Windows, Mac OS, or Linux computer with a standard installation of R and RStudio in a single step. On Windows, a desktop shortcut to the analysis script is provided. Dragging a simple text file containing analysis options onto the shortcut triggers analysis with the selected options. In addition to the standalone program, all features can also be used individually from within R. A comprehensive user guide including examples of analysis options and locus attributes is provided with the software.

### 2.6 Error, diversity, and heterozygosity calculations

Error rates for the MiSeq derived genotypes were calculated by determining the number of allelic mismatches for each sample to the known genotype of the corresponding chimpanzee (including allelic dropout, stutter sequences, PCR/sequencing artifacts, and locus amplification failure) and by dividing the total number of alleles by the number of erroneous alleles (Broquet & Petit, 2004). The expected heterozygosity (also termed gene diversity) for the sampled Gombe and GME chimpanzees was calculated from both capillary electrophoresis and MiSeq‐based microsatellite data as described in Charlesworth & Charlesworth (2010). Allelic diversity was calculated by summing the total number of unique alleles in a population.

## 3 RESULTS

### 3.1 Direct comparison of MiSeq and capillary electrophoresis‐based STR genotyping

To compare the performance of MiSeq and capillary electrophoresis side‐by‐side, we selected samples from 19 Gombe chimpanzees, who were previously genotyped by capillary electrophoresis on multiple occasions (Keele et al., 2009; Rudicell et al., 2010; Santiago et al., 2003). Testing more recently collected fecal samples that had not yet been genotyped, we used the consensus of previous genotypes at eight STR loci as the benchmark to which all MiSeq derived data were compared (Table 1). Fecal DNA was extracted, confirmed to contain more than 25 pg of chimpanzee DNA per PCR aliquot, and amplified using the same STR primers and conditions, except for the presence of MiSeq adapters versus fluorescent labels. For MiSeq sequencing, three PCR replicates were pooled, while only a single replicate was analyzed by capillary electrophoresis using both automated and manual peak calling options. The latter was performed because capillary electrophoresis analysis of pooled samples is compromised when allele peaks differ in relative height in independent PCR reactions.

**Table 1:**
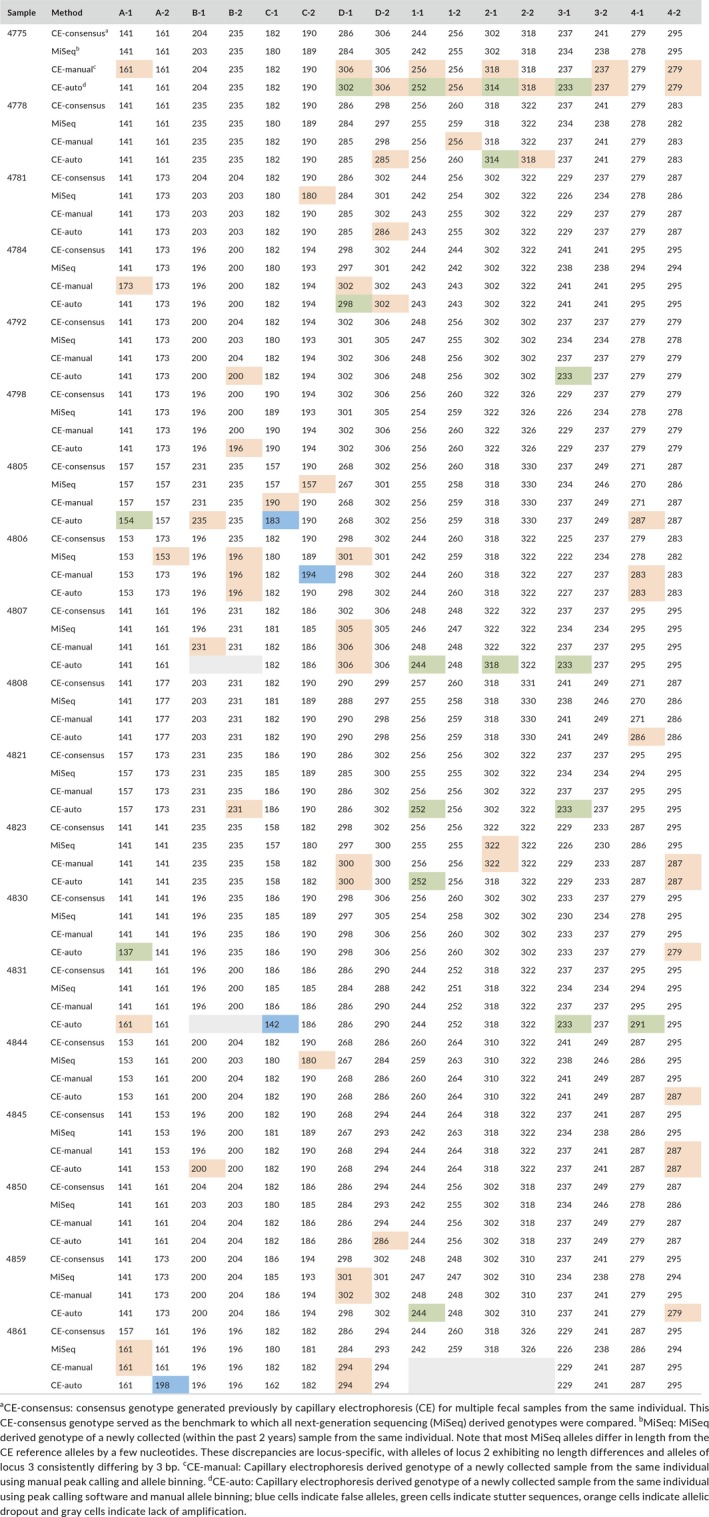
Comparison of capillary electrophoresis and MiSeq‐based genotyping results

**Figure 1:**
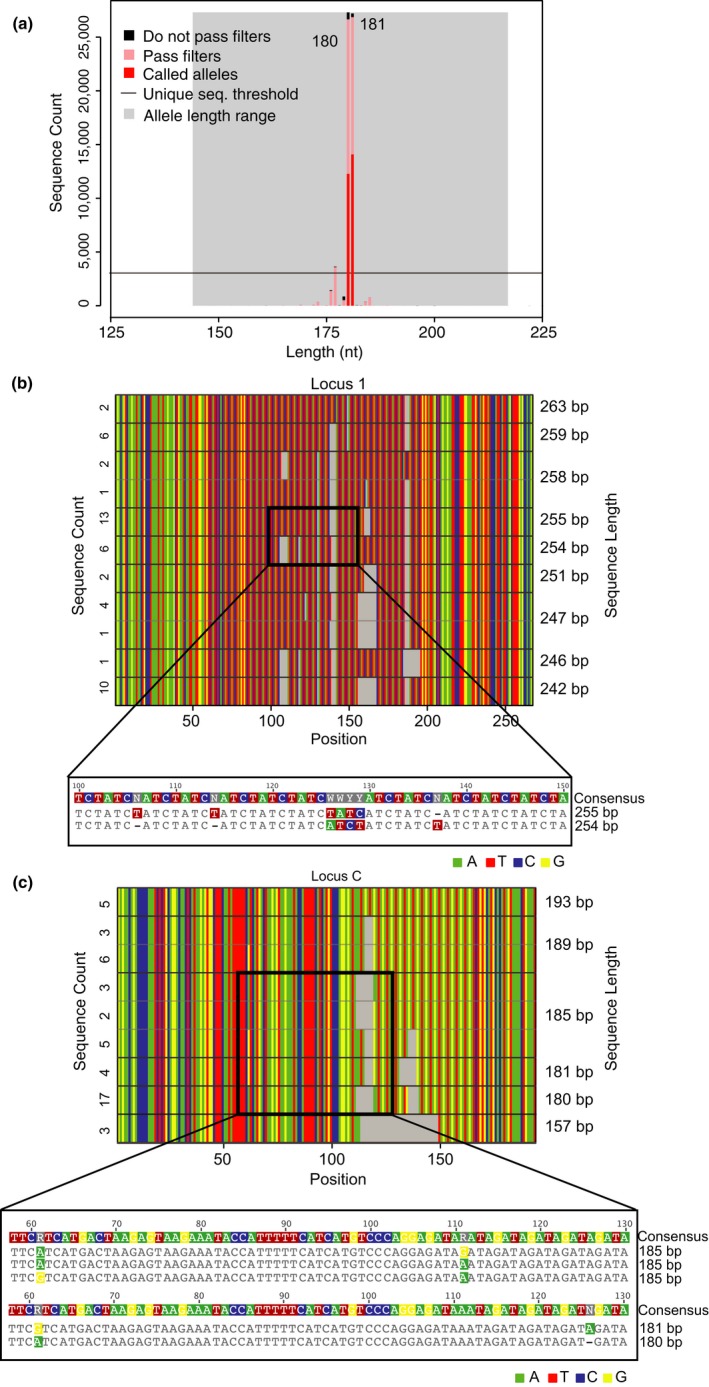
MiSeq genotyping uncovers cryptic alleles. Eight polymorphic short tandem repeat loci were amplified from the fecal DNA of 19 previously genotyped chimpanzees. (a) Histogram depicting the length (*x*‐axis) and read count (*y*‐axis) of unique sequences for one representative heterozygous locus that was previously determined to be homozygous by multiple capillary electrophoresis analyses (sample 4861, locus C, Table 1). The gray box highlights the expected locus size range. The horizontal line indicates the cutoff 5% of total filtered reads. Colored peaks indicate reads that passed the locus‐specific filters (note that peaks can be comprised of identically sized reads that differ in their sequence content). Black reads were eliminated. Pink reads appear to be locus‐specific, but did not pass the PCR artifact filters. Red reads represent the true allele sequences (180 and 181 bp in lengths, respectively). (b,c) Alignment images of locus‐specific allele sequences are shown for locus 1 (b) and locus C (c), respectively (the complete data set is shown in Table 1). Allele sequences are ordered by length (indicated in bp on the right), with the frequency with which they were found in different chimpanzees indicated on the left (the *x*‐axis indicates the position within the alignment). Nucleotides are colored as shown, with gaps in the alignment shown in gray. The insets highlight alleles that differ in their sequence content and/or length. Nucleotide substitutions are colored; dashes indicate gaps that were introduced to optimize the alignment

Using the consensus genotype of the corresponding chimpanzees for reference (Table 1), we found that MiSeq genotyping reduced the number of allelic dropouts by more than half (Table 2). This was due, at least in part, to the pooling of PCR replicates, which increased the number of alleles that were detected. However, MiSeq genotyping was also more accurate than the traditional method, which could not differentiate off‐target amplifications (Tables 1 and 2). In addition, stutter peaks were completely eliminated by the CHIIMP analysis pipleline, which was not the case for the automated capillary electrophoresis method. Although manual peak calling also eliminated stutter peaks, this was considerably more time consuming than the MiSeq approach. For the 19 samples, conventional peak calling and allele binning took 2 hr, while reviewing the bioinformatics outputs took minutes. Most importantly, MiSeq genotyping identified eight heterozygous loci that were scored as homozygous by capillary electrophoresis because of a failure to resolve minor sequence and length (1 bp) differences (Figure 1). These sequence variants were readily identified in the read histograms (Figure 1a) and their frequency identified in sequence alignments of the entire locus (Figure 1b,c). Inspection of allele lengths across all loci revealed that 24% of all MiSeq derived alleles did not differ by multiples of four, indicating frequent nucleotide insertions and deletions in the tetranucleotide repeats (Figure 1b,c).

**Table 2:** Erroneous allele calls by capillary electrophoresis and MiSeq genotyping methods

	Capillary electrophoresis (automatic)^a^	%	Capillary electrophoresis (manual)^b^	%	High‐throughput MiSeq genotyping	%
Allelic dropout	28	18^c^	21	14	10	7
Missing locus	4	3	2	1	0	0
False allele^d^	3	2	1	1	0	0
PCR stutter	18	12	0	0	0	0
Analysis time^e^	75 min		120 min		5 min	

^a^Peaks were called automatically using software. ^b^Peaks were called manually. ^c^The percentage of erroneous alleles was calculated for 152 loci by comparing the newly derived results to the reference genotypes (Table 1). ^d^Locus alleles do not match the locus primer and/or motif sequence. ^e^Hands‐on analysis time included allele length calling, binning and individual identification.

### 3.2 MiSeq genotyping uncovers increased allelic diversity and heterozygosity

To examine the true extent of allelic diversity in Gombe, we selected fecal samples from 123 chimpanzees, which included all currently living adults and juveniles, except for offspring born within the past 3 years, as well as 38 deceased individuals. All of these were previously genotyped by capillary electrophoresis on at least three occasions. Subjecting one representative fecal sample to MiSeq analysis, we confirmed 51 known alleles, but also detected 31 new alleles, which had previously gone unrecognized due to size (1 bp) or nucleotide sequence differences (Tables 3 and Supporting Information Figure S2). Such cryptic alleles were detected for all eight STR loci, increasing allelic diversity by an average of 1.6‐fold per locus. Nearly half of all previously reported alleles had closely related length or sequence variants (Table 4).

**Table 3:** Increased allelic and gene diversity as detected by MiSeq STR genotyping

Locus	Number of alleles^a^			Gene diversity^b^	
	CE	MiSeq	Cryptic^c^	CE	MiSeq
A	6	7	1	0.74	0.74
B	5	7	2	0.79	0.81
C	5	10	5	0.70	0.83
D	7	13	6	0.80	0.88
1	9	16	7	0.80	0.86
2	7	9	2	0.75	0.75
3	7	14	7	0.71	0.83
4	5	6	1	0.72	0.80
Total/mean	51	82	31	0.75	0.81

*Notes*

CE: capillary electrophoresis.

^a^Number of alleles at eight STR loci determined for 123 Gombe chimpanzees (Supporting Information Table S2). ^b^Nine individuals were excluded from heterozygosity calculations because they had incomplete CE genotypes. ^c^Alleles newly discovered by MiSeq genotyping.

**Table 4:** Allelic sequence and length differences uncovered by MiSeq‐based genotyping

Locus	Cryptic allele^a^	Number of apes carrying allele	Substitutions (identical length)	Indels (identical length)	Indels (1 bp length difference)	Mendelian inheritance
A	157‐b	3	2			Yes
B	204‐a	14			1	Yes
B	231‐b	2	1			Yes
C	181‐a	10			1	Yes
C	181‐b	1^b^	1			NA
C	185‐b	20	1			Yes
C	185‐c	11	1			Yes
C	189‐b	35	1			Yes
D	285‐a	8	3		1	Yes
D	297‐b	8		2		Yes
D	297‐c	7	1			Yes
D	300‐a	14	1		1	Yes
D	301‐b	7	3			Yes
D	305‐b	11	1			Yes
1	246‐a	10			5	Yes
1	247‐b	4		2		Yes
1	250‐a	2			5	Yes
1	254‐a	27			1	Yes
1	258‐a	6			5	Yes
1	258‐b	3			3	Yes
1	266‐b	2		4		Yes
2	310‐b	1^b^	1			NA
2	326‐b	6	1			NA
3	226‐b	1^b^		2		NA
3	230‐b	1^b^	3			NA
3	234‐b	25		2		Yes
3	234‐c	10	3	2		Yes
3	234‐d	4		2		Yes
3	238‐b	3		2		NA
3	246‐b	7		2		Yes
4	294‐a	38	3	2		Yes

^a^Cryptic alleles were compared to the most abundant allele of the same or similar length. ^b^Alleles found in only one chimpanzee were confirmed by repeat amplification and sequencing.

**Figure 2:**
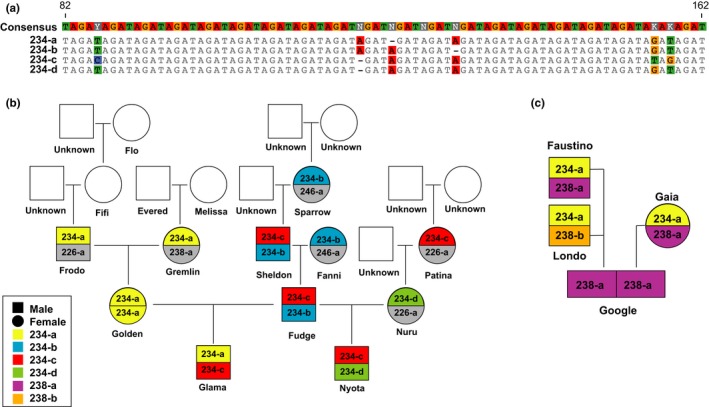
MiSeq genotyping uncovers increased allelic diversity and heterozygosity. (a) Alignment of four locus 3 alleles that are of identical length (234 bp), but differ in sequence content. Nucleotide substitutions are colored; dashes indicate single nucleotide insertions and deletions (b) Mendelian inheritance of allele 234 for a group of related chimpanzees. Fathers and mothers are shown as squares and circles, respectively, with offspring connected by vertical lines. Both alleles are shown for each animal, with the four allelic variants highlighted in different colors. Individuals of unknown identity or genotype are left blank. (c) Increased allelic diversity resolves a previously ambiguous paternity determination. Two potential fathers with identical allele lengths (238 bp) can now be distinguished based on differences in allele sequence content (238‐a and 238‐b). As the offspring is homozygous for allele 238‐a, the male with allele 238‐b can be excluded as a father

Although the great majority of the newly identified alleles were found in multiple individuals, we wanted to validate their authenticity by demonstrating inheritance. As paternity and kinship relationships are known for most Gombe chimpanzees, we were able to trace the majority of the newly identified allelic variants from parents to their offspring. For example, Locus 3 includes four alleles that are identical in size (234 bp) but differ by up to three substitutions and two single nucleotide insertions and deletions (Figure 2a). Alleles 234‐a, 234‐b, 234‐c, and 234‐d were found in 80, 25, 10 and 4 chimpanzees, respectively, including several parent‐offspring triads (Figure 2b). Overall, we were able to document inheritance for 25 (81%) of the 31 new alleles. For the remaining six, existing pedigree information was insufficient, and their existence was thus confirmed by sequencing at least two independent PCR amplicons (Table 4).

The newly identified alleles revealed that over a quarter of genotypes at loci previously assigned as homozygous (60 of a total of 228) were in fact heterozygous (Supporting Information Table S2). This increased allelic diversity resolved one case of an ambiguous paternity determination. Using the standard eight STR loci, we were previously unable to identify the father of one infant (Google) because two candidate males (Faustino and Londo) had the identical genotype at all eight STR loci (Walker et al., 2017). Using the new genotypes, we were able to exclude Londo and confirm Faustino as a father by revealing differences at one locus (Figure 2c). Although Faustino was identified as the correct father at the time by genotyping 10 additional loci using capillary electrophoresis (Walker et al., 2017), this would not have been necessary had the increased allelic diversity been known. Thus, MiSeq genotyping revealed much greater allelic and microsatellite gene diversity in Gombe than previously appreciated, thus increasing the analytical potential of the existing STR loci.

### 3.3 MiSeq genotyping based individual identification

**Figure 3:**
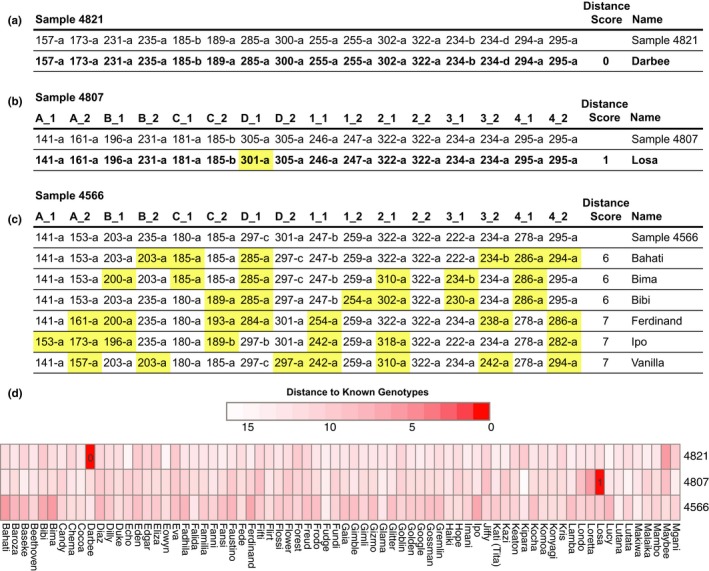
Individual identification based on MiSeq genotyping. (a–c) Genotypes of newly collected samples (top) are compared to the genotypes of known community members, with the closest match listed below (based on descending distance scores). Genotypes that differ by fewer than four alleles are indicated in bold because they represent likely matches. Differences are highlighted in yellow. (d) Heatmap showing the relative similarity of sample genotypes (rows) with genotypes of known individuals (columns) based on distance scores. Dark red cells indicate likely matches

As chimpanzee communities are often studied longitudinally, we added an individual identification tool to the analysis platform. This tool compares the genotype of every new sample with all previously characterized genotypes and generates a distance score to indicate their relative similarity. For example, samples with a distance score of 0 match at all loci, while samples with a distance score of 2 differ by two alleles. We then used this approach to characterize the same 19 newly genotyped samples (Table 1) as well as five samples from infants with unknown genotypes. To account for allelic dropout, a distance score of up to 3 was allowed. The results revealed accurate individual identification for all samples from previously genotyped chimpanzees. Of the 19 samples, eight exhibited a perfect match across all loci (Figure 3a), while 11 others had distance scores of 1–3, which were consistent with allelic dropout (Figure 3b). However, five samples with distance scores of 5–7 could not be assigned to known individuals (Figure 3c), and a review of field notes revealed that they were all collected from new infants. A heatmap allowed the quick identification of very close (4821, 4807) and very distant (4566) matches (Figure 3d). Thus, the individual identification tool detected previously determined genotypes with reasonable accuracy.

### 3.4 STR genotyping of multiplexed samples

Chimpanzees in the Greater Mahale Ecosystem in Tanzania occupy a large home range, live at low population densities, and face extreme seasonal changes (Moore, 1996; Ogawa, Idani, & Kanamori, 1999; Schoeninger, Moore, & Sept, 1999). Thus, these “savanna chimpanzees” live under ecologically more challenging conditions than their forest‐dwelling counterparts, and with the exception of the Issa community, are not habituated. As a result, fecal collections, sample transport, and storage are logistically more difficult, which can result in reduced amounts of collected material and/or partially degraded host DNA. To test the suitability of MiSeq genotyping for such samples, we selected 12 previously characterized chimpanzee fecal specimens from the Issa Valley (Rudicell et al., 2011) and re‐genotyped them using both singleplex and multiplex locus amplification. Singleplex PCR was performed as in Gombe, while multiplex PCR was carried out in two steps as previously described (Arandjelovic et al., 2009). First, PCR primers for four loci were pooled and used to amplify fecal DNA in two (rather than eight) reactions (one‐step multiplex product). Second, aliquots of this first round PCR were then used in a second round of PCR to amplify each of the eight STR loci separately (two‐step multiplex product). Three pooled replicates of both one‐step and two‐step multiplexed products were sequenced and compared to the previously determined genotypes (Supporting Information Table S3). Although the overall amplification efficiency was lower than originally reported (most likely due to repeated freezing and thawing of the 7–8 year‐old samples), one‐step multiplexing performed as well as singleplex PCR, but used only a quarter of the fecal DNA (Table 5). Two‐step multiplexing detected slightly more alleles, but not surprisingly, also resulted in an increased number of stutter sequences and other PCR artifacts. Thus, one‐step multiplexing required less starting material and was also more cost efficient because the combined loci were sequenced in a single MiSeq run (and were subsequently de‐multiplexed bioinformatically).

**Table 5:** MiSeq genotyping of singleplex and multiplex amplified STR loci

	Singleplex PCR	%	One‐step multiplex PCR	%	Two‐step multiplex PCR	%
Allele detection	130	68^a^	130	68	147	77
Incorrect allele	1	0.5	1	0.5	4	2.1
PCR stutter	1	0.5	1	0.5	4	2.1
DNA Input	24 μl		6 μl		6 μl	

a Of a total of 192 alleles analyzed for 12 GME chimpanzees.

**Figure 4:**
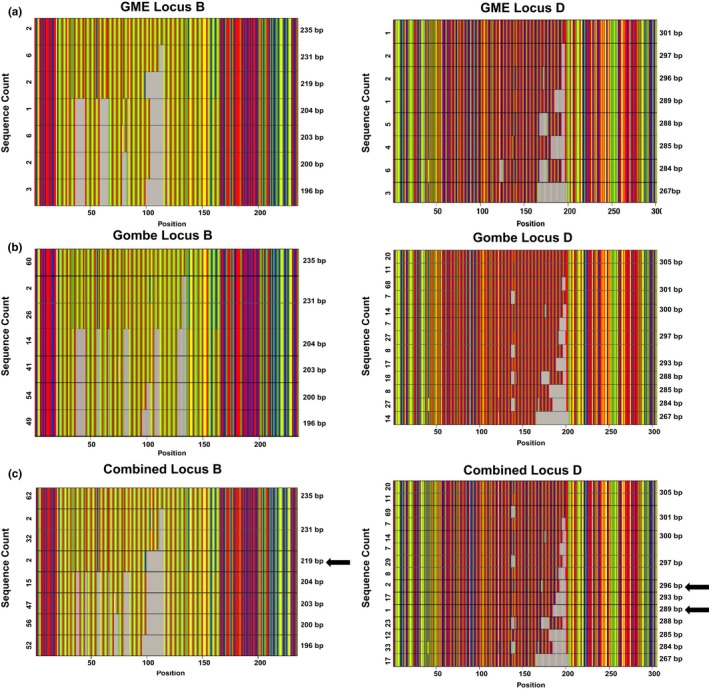
Comparison of MiSeq genotypes across chimpanzee communities. Alignment images of locus‐specific allele sequences are shown for chimpanzees from the Greater Mahale Ecosystem (GME) and Gombe. Two representative loci (locus B on the left; locus D on the right) are shown for (a) 12 chimpanzees from the GME (Supporting Information Table S3), (b) 123 chimpanzees from Gombe (Supporting Information Table S2), and (c) a combination of both. Allele sequences are ordered by length (indicated in base pairs on the right), with the frequency with which they were found in different chimpanzees indicated on the left (the *x*‐axis indicates the position within the alignment). Nucleotides are colored as indicated, with alignment gaps shown in gray. Arrows indicate alleles that are unique to the GME samples

MiSeq genotyping also allowed us to compare the allelic diversity in Gombe and the GME. Figure 4 depicts such an analysis for locus B and D, highlighting alleles that were only found in GME chimpanzees. Comparing all eight STR loci, we found ten alleles in only 12 GME chimpanzees that were absent from the 123 genotyped Gombe individuals, six of which represented alleles previously missed in the GME due to sequence and length differences. Although the mean expected heterozygosity value for the GME chimpanzees (0.743) was lower than that for Gombe (0.812), this is likely due to the small sample size and the fact that all 12 individuals were sampled at a single location in Issa Valley (Rudicell et al., 2011). Additional samples from more diverse locations in the GME are needed to compare the genetic diversity of this population to that of Gombe and other field sites.

## 4 DISCUSSION

**Figure 5:**
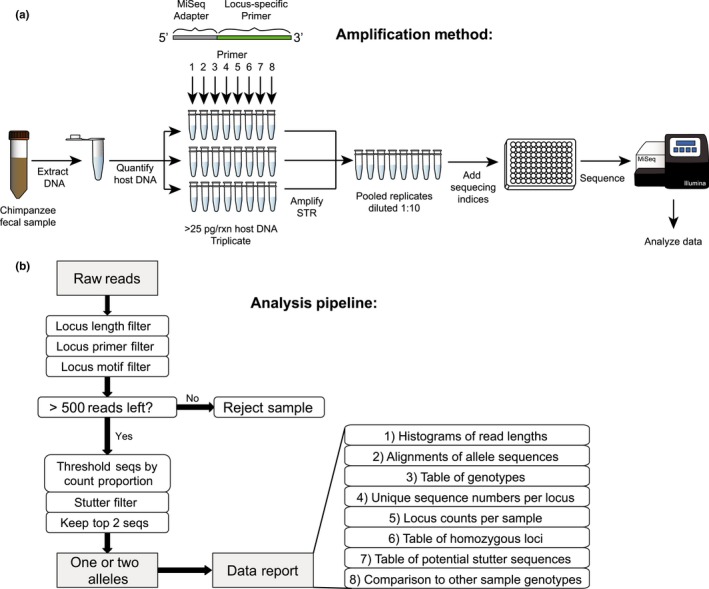
MiSeq‐based short tandem repeat (STR) genotyping of wild chimpanzees. (a) Schematic representation of singleplex STR amplification and MiSeq sequencing of chimpanzee fecal DNA. (b) Schematic representation of the CHIIMP analysis pipeline with decision tree and downstream data reports

Over the past two decades, microsatellite analyses have been an integral part of studies of wild chimpanzees, providing insight into their evolution, population genetics, behavior, disease association and social structure (Barbian et al., 2018; Becquet, Patterson, Stone, Przeworski, & Reich, 2007; Keele et al., 2009; Langergraber, Mitani, & Vigilant, 2007; Moeller et al., 2016; Rudicell et al., 2010; Santiago et al., 2003; Vigilant, Hofreiter, Siedel, & Boesch, 2001; Walker et al., 2017; Wroblewski et al., 2015). However, traditional genotyping methods are cumbersome, imprecise and investigator/platform dependent, due to the use of capillary electrophoresis to determine the length of STR loci. Here, we report a high‐throughput MiSeq‐based approach, which represents a marked improvement, because it is faster, more accurate and able to detect the full extent of allelic diversity in a population. Moreover, it includes a new analysis platform, CHIIMP, which not only automates the conversion of raw MiSeq data into multilocus genotypes, but also implements a number of quality control measures that improve genotyping accuracy (Figure 5). Of note, CHIIMP has been designed for maximal customization. While analysis of pedigreed fecal samples from chimpanzees allowed rigorous validation, the pipeline is not limited to a particular species or sample type.

### 4.1 Improved accuracy of MiSeq‐based genotyping

Sequence‐based genotyping methods not only determine the length of STR loci, but also reveal their sequence content, and thus have the potential to detect a greater number of distinct alleles than capillary electrophoresis. Indeed, such genotyping of Atlantic cod and muskrats revealed high proportions of cryptic alleles, ranging from 32% to 44% (Darby et al., 2016; Vartia et al., 2016). In light of these data, our discovery of 38% new alleles (31 of 82) in Gombe is not surprising (Table 3). However, this finding suggests that existing STR data vastly underestimate the diversity of microsatellite sequences in wild chimpanzees, not only in Gombe but also in other populations. New alleles were found for all loci, with some comprising twice as many variants as previously observed (Table 3), which will undoubtedly add to the statistical power of future analyses. However, any new allele will have to be examined carefully by repeat amplification and sequencing, unless it can be validated by pedigree analysis. In our dataset, a minor fraction of “new” alleles was found to represent PCR and/or sequencing artifacts that exceeded the 33% threshold for heterozygous alleles. Repeat amplification of these alleles resolved all sequencing artifacts.

Comparison of the MiSeq data to validated reference genotypes also allowed us to assess the error rate of the new approach. After implementation of all filters, CHIIMP eliminated 98% of stutter sequences and 100% of off‐target amplicons. Among the samples tested, true alleles, allelic dropouts, and false alleles were detected with a frequency of 96%, 7%, and 0%, respectively. These data are comparable to MiSeq derived genotyping results for wild‐living brown bears, where true alleles, allelic dropouts, and false alleles were detected with a frequency of 93%, 0.4% and 0.05% for tissues, and 80%, 14% and 1% for fecal samples, respectively (De Barba et al., 2017). Although our overall error rate of 3.3% is slightly higher than the 2.1% error rate reported for a MiSeq genotyping study of laboratory‐raised (pedigreed) fish (Zhan et al., 2017), this is not surprising as the latter study examined freshly extracted tissue DNA.

As noninvasively collected samples frequently contain diluted and/or degraded host DNA, they are genotyped using multiple PCR reactions to guard against the selective loss of alleles (allelic dropout). Loci are only considered homozygous if they can be confirmed in multiple PCR reactions (Morin et al., 2001; Taberlet et al., 1996). Capillary electrophoresis requires that these replicates are run independently to distinguish true alleles from nonspecific signal, often more than tripling the amount of time and effort required to genotype a single sample. In contrast, MiSeq genotyping can be performed after combining the products of multiple PCR reactions. Although the quality of the input DNA remains the same, MiSeq genotyping of pooled PCR replicates reduces the frequency of allelic dropout and thus renders the resulting genotypes more accurate. However, amplicon pooling foregoes data from repeat analyses, which are used by some as a measure of DNA quality and/or data reliability (Taberlet et al., 1996).

Once MiSeq data files are imported into the CHIIMP platform, the program calls alleles automatically, thus saving days of hands‐on work. While automated allele calling has been reported previously (De Barba et al., 2017; Suez et al., 2016; Zhan et al., 2017), CHIIMP includes downstream analyses, such as alignments of allele sequences or flagging loci that may contain contaminants, which provide important additional quality control measures. In contrast to previous studies, CHIIMP also retains nonrepeat regions (Suez et al., 2016), which can contribute to allelic diversity, and does not require the presence of stutter sequences for allele calling, which may not be sufficiently abundant under conditions of low coverage (De Barba et al., 2017). Finally, CHIIMP reports both allele length and sequence content and is thus designed to detect minor length and sequence differences by including sequence‐specific allele names and generating locus‐specific sequence alignments (Fig. 4 and Supporting Information Figure S1). To guide subsequent analyses, we have also added features that flag potentially problematic alleles and standardize allele naming. CHIIMP thus represents the most comprehensive analysis platform yet to ensure the accuracy of MiSeq‐based genotyping results.

### 4.2 Multiplexing improves MiSeq genotyping efficiency and reduces cost

The Illumina MiSeq v2 500 sequencing kit has an output of ~25 million reads per run, thus allowing the multiplexing of many samples, the number of which depends on the desired read depth. Comparing read depths per allele, we found that a cutoff of 500 reads yielded the most accurate results for our dataset. This value is higher than the 50 read cutoff used previously to genotype laboratory raised fish (Zhan et al., 2017). However, the latter study used high‐quality tissues rather than fecal samples for analysis. To determine the sources of allele‐calling errors, we did not multiplex samples from Gombe chimpanzees. However, we tested multiplexing using samples from GME chimpanzees and confirmed that this approach yields accurate results. Although primer incompatibilities allowed the combination of only four loci, this number can be significantly increased with additional primer design. For example, a recent study genotyped bear fecal DNA by multiplexing 14 loci (De Barba et al., 2017). Pooling amplicons from multiple loci after singleplex PCR can circumvent the need for specialized primer design, as the maximum number of pooled loci for any given sample is limited only by the desired read depth. Moreover, barcoding of individual samples allows their combination in sequencing reactions, thus further increasing sequencing efficiency and throughput (Farrell et al., 2016).

MiSeq genotyping is expensive, but these costs decrease with sample numbers. Capillary electrophoresis is undoubtedly cheaper when only a small number of samples has to be analyzed; however, MiSeq sequencing becomes increasingly more cost‐effective with multiplexing and analyses of pooled replicates (Darby et al., 2016). The costs of MiSeq genotyping three replicates of 96 samples multiplexed at four loci would roughly be equivalent to analyzing the same multiplexed samples via capillary electrophoresis, because the latter cannot analyze pooled replicates. While this estimate only considers genotyping supplies, labor to manually analyze samples is not included. In addition, the improved accuracy has downstream cost advantages as fewer repeat analyses would have to be performed.

### 4.3 Effective sharing of MiSeq genotyping data

A direct comparison of MiSeq and capillary electrophoresis derived alleles revealed consistent length differences of one to three nucleotides, the number of which was locus specific (Tables 1 and Supporting Information Figure S3). For Gombe samples, locus 3 alleles derived by capillary electrophoresis were always three nucleotides longer than the corresponding MiSeq alleles (Table 1). However, for the GME samples, the same alleles were all one nucleotide shorter than the MiSeq alleles (Supporting Information Table S3). This is as expected as the capillary electrophoresis data were generated on different platforms. However, this also means that a simple conversion of existing capillary electrophoresis to MiSeq data will generally not be possible. In contrast, MiSeq genotyping generates unambiguous alleles that can be compared across multiple studies and field sites (Figure 4). In the future, it will thus be possible to compare STR genotypes across different chimpanzee populations, such as those in Gombe and the GME, as the use of different sequencing equipment will no longer confound these analyses.

### 4.4 Versatility of the CHIIMP analysis platform

To increase its utility, we designed the CHIIMP analysis platform to be versatile. STR locus attributes, such as the expected length range, primer sequences, and repeat motifs, as well as all thresholds for allele calling, can be customized. For example, analysis of loci with dinucleotide repeats may require a lower threshold for stutter peaks, as these are more susceptible to polymerase slippage (Guichoux et al., 2011). Similarly, locus length ranges can be expanded or contracted, depending on the rate of off‐target amplicons. The CHIIMP analysis pipeline also includes tools that facilitate iterative improvements for new applications (Supporting Information Figure S1). For example, the program provides a heatmap that indicates the number of unique sequences that pass all filters. If that number is too high, thresholds can be adjusted to remove stutter peaks, off‐target amplicons, and/or PCR errors. In addition, the distribution of loci is visualized, which can be used to reveal contamination in singleplexed samples or identify poorly performing primers in multiplexed samples (Supporting Information Figure S1g). For potentially problematic alleles, CHIIMP generates histograms that provide information concerning their length and relative abundance. All of these tools can be used to adapt the platform to additional STR loci and/or host species.

The length of STR loci suitable for sequence‐based genotyping depends on the sequencing chemistry. We used Illumina v2 technology, which has maximum read lengths of 500 nucleotides. MiSeq sequences are most often generated using paired‐end reads, with a maximum read length of 250 nucleotides in each direction. For STR genotyping, locus sequences must span the repeat motif region, as assembly of shorter reads could result in misalignments. To accommodate loci of greater than 250 bp length, we opted to only use forward reads for analysis. Although the sequencing kit could theoretically accommodate fragments of up to 500 nucleotides, we found that the quality of reads (*Q* scores) decreased significantly after 400 cycles. Given that the longest locus in our panel spanned 357 nucleotides, we used 375 cycles in the forward direction. Illumina v3 sequencing chemistry has a 600‐cycle limit, which may accommodate loci of up to 500 bp, but this would have to be determined experimentally. The majority of microsatellite loci are shorter than this length.

As STR genotyping transitions from capillary electrophoresis to sequence‐based approaches, it will be necessary to standardize allele nomenclature, as has already been suggested for human forensics (Gelardi, Rockenbauer, Dalsgaard, Børsting, & Morling, 2014; Parson et al., 2016). At a minimum, allele names will have to incorporate the length and unique sequence content for each allele (Darby et al., 2016). In our study, we added an alphabetical identifier (‐a, ‐b, ‐c, etc.) to differentiate identically sized alleles that differed in their sequence (Supporting Information Table S2). As it is impossible to capture all allele attributes in a single name, it may become necessary to establish databases that link allele identifiers to their respective sequences. CHIIMP is designed to allow users to supply a spreadsheet of allele names and sequences, and thus guarantees consistent nomenclature across experiments. As MiSeq genotyping is adapted to additional projects, standardized allele designations will become necessary to ensure consistent nomenclature across studies.

### 4.5 Conclusions

Genetic study of wild primates and other endangered species has been shown to provide more accurate information concerning the size, structure, distribution, and dynamics of populations than observational studies. However, genotyping can be prohibitively expensive given the large numbers of samples that are required for such analyses. The MiSeq‐based genotyping platform provides a new approach that drastically reduces time and labor, while providing more accurate and informative genotypes compared to capillary electrophoresis. This will allow much faster and more streamlined analysis of samples that are necessary for censusing and monitoring of nonhabituated populations in addition to revealing previously inaccessible allelic diversity. The CHIIMP platform has been designed to be adaptable to additional loci and/or species. This allows the study of group membership, dispersal, gene flow, and association patterns for a multitude of wildlife species with broad conservation and biological implications.

## CONFLICT OF INTEREST

The authors declare no competing financial interests.

## AUTHOR CONTRIBUTIONS

All authors contributed to the acquisition, analysis, and interpretation of the data. H.J.B., A.J.C. and B.H.H. conceived, planned and executed the study; H.J.B., A.N.A., R.M.R., M.S.G. and Y.L. performed STR locus amplifications and data analyses; H.J.B. and A.J.C. developed the CHIIMP analysis pipeline; A.G.S., A.L.S., and F.B.R. optimized the MiSeq sequencing approach; D.M., E.V.L, F.A.S., A.K.P., and A.E.P. conducted or supervised field work; A.J.C., E.E.W, and P.M.S. performed allelic diversity and parentage analyses; H.J.B., A.J.C., R.M.R. and B.H.H coordinated the contributions of all authors and wrote the manuscript.

## DATA ACCESSIBILITY

STR sequences are archived in the NCBI Sequence Read Archive (SRA) under https://www.ncbi.nlm.nih.gov/bioproject/PRJNA434411. Preprocessed sequence data, analysis software and supporting R code are archived on Dryad https://doi.org/10.5061/dryad.59j3974. Ongoing Software Development is available at https://github.com/ShawHahnLab/chiimp and supporting R code at https://github.com/ShawHahnLab/chiimp-paper.
