# Supplementary material for: CHIIMP: An automated high‐throughput microsatellite genotyping platform reveals greater allelic diversity in wild chimpanzees
Source: Ecol Evol. 2018 Jul 16;8(16):7946–63. doi: 10.1002/ece3.4302 (PMC6145012; doi:10.1002/ece3.4302)

(a) Genotype Summary

Loci: A-D

Sample	A_1	A_2	B_1	B_2	C_1	C_2	D_1	D_2	Distance	Name
4566	141-a	153-a	203-a	235-a	180-a	185-a	297-c	301-a		
4634	141-a	161-a	200-a	235-a	185-b	189-b	305-a	305-a		
4704	161-a	161-a	200-a	235-a	180-a	180-a	297-b	301-a		
4775	141-a	161-a	203-a	235-a	180-a	189-b	284-a	305-b	0	Gremlin
4778	141-a	161-a	235-a	235-a	180-a	189-b	284-a	297-b	0	Gaia
4781	141-a	173-a	203-a	203-a	180-a	180-fdd1c6	284-a	301-a	1	Glitter
4784	141-a	173-a	196-a	200-a	180-a	193-a	297-a	301-a	0	Flirt
4792	141-a	173-a	200-a	203-a	180-a	193-a	301-a	305-b	0	Golden
4798	141-a	173-a	196-a	200-a	189-b	193-a	301-a	305-b	0	Fanni
4805	157-a	157-a	231-a	235-a	157-a	157-489d0d	267-a	301-a	1	Chema
4806	153-a	153-a	196-a	196-a	180-a	189-b	301-a	301-a	3	Ipo
4807	141-a	161-a	196-a	231-a	181-a	185-b	305-a	305-a	1	Losa
4808	141-a	177-a	203-a	231-a	181-a	189-a	288-a	297-a		
4813	141-a	173-a	196-a	231-a	180-a	193-a	301-a	305-b		
4821	157-a	173-a	231-a	235-a	185-b	189-a	285-a	300-a	0	Darbee
4822	141-a	141-a	200-a	235-a	180-a	185-a	288-a	300-a		
4823	141-a	141-a	235-a	235-a	157-a	180-a	297-a	300-a	0	Eliza
4830	141-a	141-a	196-a	235-a	185-b	189-a	297-a	305-a	1	Edgar
4831	141-a	161-a	196-a	200-a	185-a	185-c	284-a	288-a	0	Sheldon
4844	153-a	161-a	200-a	203-a	180-a	180-fdd1c6	267-a	284-a	1	Kati (Tita)
4845	141-a	153-a	196-a	200-a	181-a	189-b	267-a	293-a	0	Kazi
4850	141-a	161-a	203-a	203-a	180-a	185-a	284-a	293-a	0	Gimli
4859	141-a	173-a	200-a	204-a	185-c	193-a	301-b	301-a	1	Zeus
4861	161-a	161-a	196-a	196-a	180-a	181-a	284-a	293-a	2	Nasa

Fig. S1

(b) Inter-Sample Distance Matrix

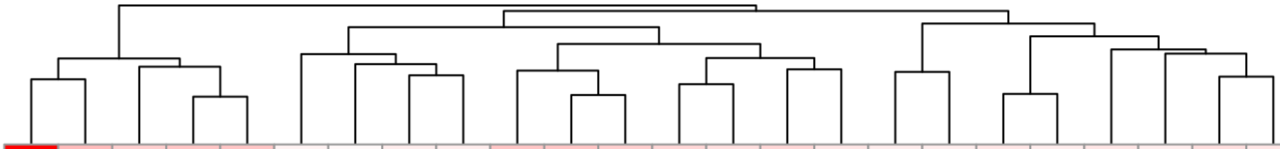

	4792	4859	4784	4798	4813	4831	4861	4844	4845	4781	4775	4850	4704	4778	4566	4806	4805	4808	4822	4823	4807	4821	4634	4830
4792		7	9	8	8	14	15	13	13	8	8	9	10	11	10	12	13	13	12	13	12	13	11	12
4859	7		8	10	10	12	14	13	12	10	11	12	12	12	12	13	14	14	14	15	13	13	12	13
4784	9	8		9	9	10	11	12	10	9	11	12	12	12	12	11	15	13	11	12	13	12	13	12
4798	8	10	9		5	11	13	13	11	9	12	14	11	11	11	10	15	15	12	13	13	13	10	11
4813	8	10	9	5		11	13	14	13	8	10	12	11	13	11	12	14	14	12	12	12	11	11	9
4831	14	12	10	11	11		10	11	9	12	10	11	11	11	12	12	15	13	8	13	11	12	12	12
4861	15	14	11	13	13	10		10	9	11	10	9	12	10	14	10	14	12	13	13	13	15	15	15
4844	13	13	12	13	14	11	10		8	10	10	10	11	10	10	12	14	13	11	12	13	14	14	15
4845	13	12	10	11	13	9	9	8		11	9	10	11	10	11	9	12	11	10	12	10	14	13	13
4781	8	10	9	9	8	12	11	10	11		8	6	10	10	9	10	13	13	11	11	13	13	12	12
4775	8	11	11	12	10	10	10	10	9	8		5	8	5	9	10	12	11	10	11	12	12	9	11
4850	9	12	12	14	12	11	9	10	10	6	5		10	8	11	11	12	11	11	12	12	13	10	12
4704	10	12	12	11	11	11	12	11	11	10	8	10		7	9	10	11	14	10	11	12	12	10	12
4778	11	12	12	11	13	11	10	10	10	10	5	8	7		9	8	12	12	11	10	12	13	10	13
4566	10	12	12	11	11	12	14	10	11	9	9	11	9	9		8	13	14	9	10	11	13	13	12
4806	12	13	11	10	12	12	10	12	9	10	10	11	10	8	8		13	15	13	14	13	15	14	14
4805	13	14	15	15	14	15	14	14	12	13	12	12	11	12	13	13		9	13	12	14	12	13	14
4808	13	14	13	15	14	13	12	13	11	13	11	11	14	12	14	15	9		12	12	13	13	13	13
4822	12	14	11	12	12	8	13	11	10	11	10	11	10	11	9	13	13	12		5	12	10	11	10
4823	13	15	12	13	12	13	13	12	12	11	11	12	11	10	10	14	12	12	5		12	10	13	10
4807	12	13	13	13	12	11	13	13	10	13	12	12	12	12	11	13	14	13	12	12		12	11	11
4821	13	13	12	13	11	12	15	14	14	13	12	13	12	13	13	15	12	13	10	10	12		11	10
4634	11	12	13	10	11	12	15	14	13	12	9	10	10	10	13	14	13	13	11	13	11	11		8
4830	12	13	12	11	9	12	15	15	13	12	11	12	12	13	12	14	14	13	10	10	11	10	8	

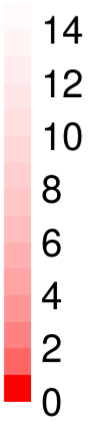

(c) Identification with Known Genotypes

Loci: A-D; Samples 4566-4781

A_1	A_2	B_1	B_2	C_1	C_2	D_1	D_2	Distance	Name
**Sample 4566**									
141-a	153-a	203-a	235-a	180-a	185-a	297-c	301-a		
141-a	153-a	203-a	203-a	185-a	185-a	285-a	297-c	6	Bahati
141-a	153-a	200-a	203-a	185-a	185-a	285-a	297-c	6	Bima
141-a	153-a	203-a	235-a	185-a	189-a	285-a	297-a	7	Bibi
141-a	161-a	200-a	235-a	180-a	193-a	284-a	301-a	7	Ferdinand
153-a	173-a	196-a	235-a	180-a	189-b	297-b	301-a	7	Ipo
141-a	157-a	203-a	203-a	180-a	185-a	297-c	297-a	7	Vanilla
**Sample 4634**									
141-a	161-a	200-a	235-a	185-b	189-b	305-a	305-a		
161-a	177-a	200-a	235-a	180-a	189-b	305-a	305-a	6	Konyagi
141-a	161-a	200-a	235-a	180-a	180-a	301-a	305-a	7	Kocha
161-a	177-a	235-a	235-a	180-a	189-b	305-a	305-a	7	Komoa
**Sample 4704**									
161-a	161-a	200-a	235-a	180-a	180-a	297-b	301-a		
141-a	161-a	200-a	235-a	180-a	180-a	284-a	301-a	4	Faustino
**Sample 4775**									
141-a	161-a	203-a	235-a	180-a	189-b	284-a	305-b		
**141-a**	**161-a**	**203-a**	**235-a**	**180-a**	**189-b**	**284-a**	**305-b**	**0**	**Gremlin**
**Sample 4778**									
141-a	161-a	235-a	235-a	180-a	189-b	284-a	297-b		
**141-a**	**161-a**	**235-a**	**235-a**	**180-a**	**189-b**	**284-a**	**297-b**	**0**	**Gaia**
**Sample 4781**									
141-a	173-a	203-a	203-a	180-a	180-fdd1c6	284-a	301-a		
**141-a**	**173-a**	**203-a**	**203-a**	**180-a**	**189-b**	**284-a**	**301-a**	**1**	**Glitter**

(d) Distance Matrix for Known Genotypes

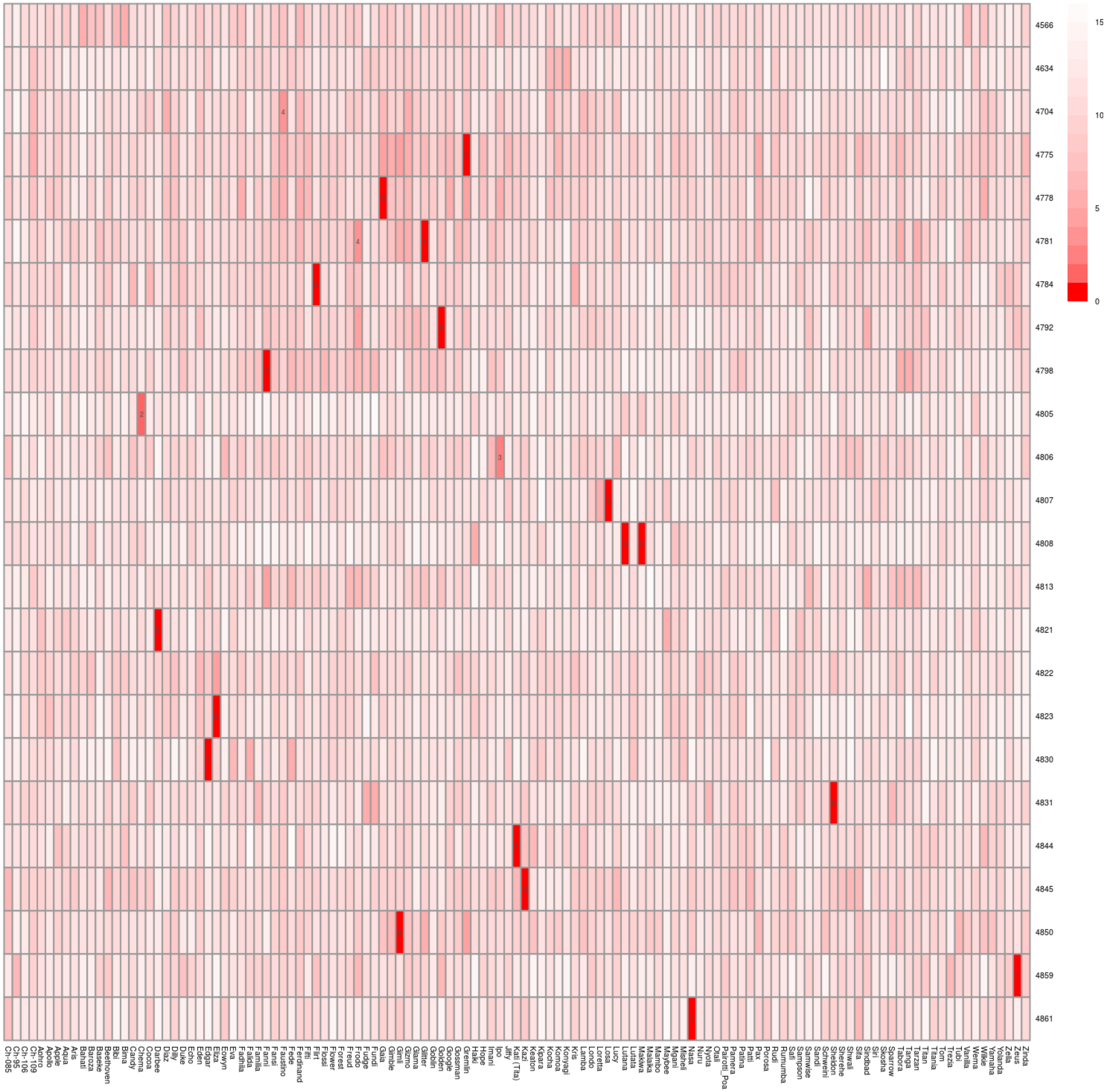

(e) Flagged Values

Loci subjected to stutter filter

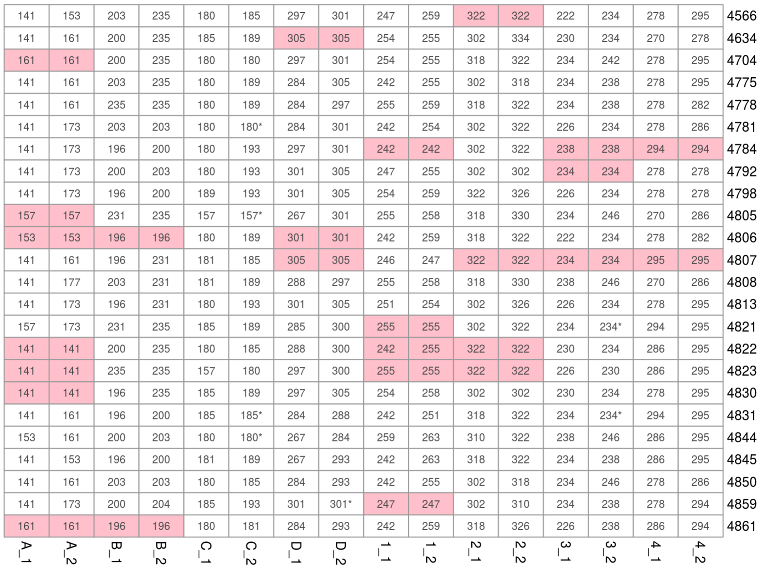

Loci with more than two prominent sequences

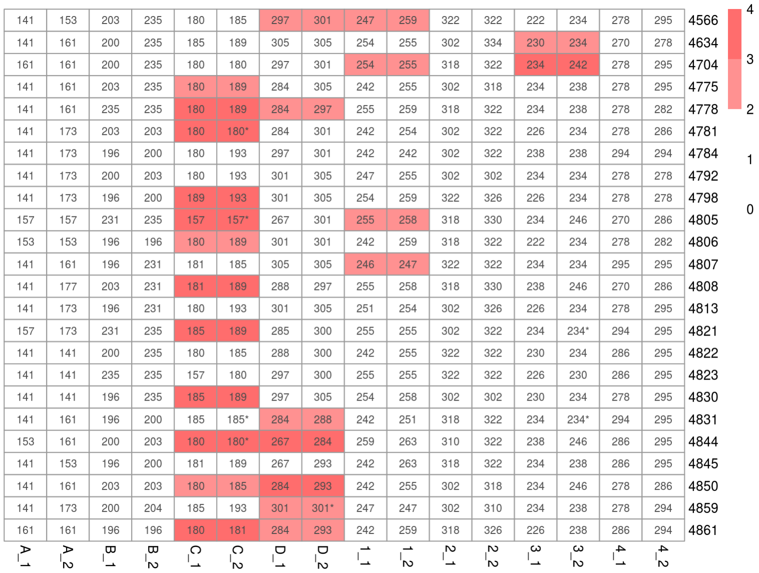

Proportion of allele-matching reads

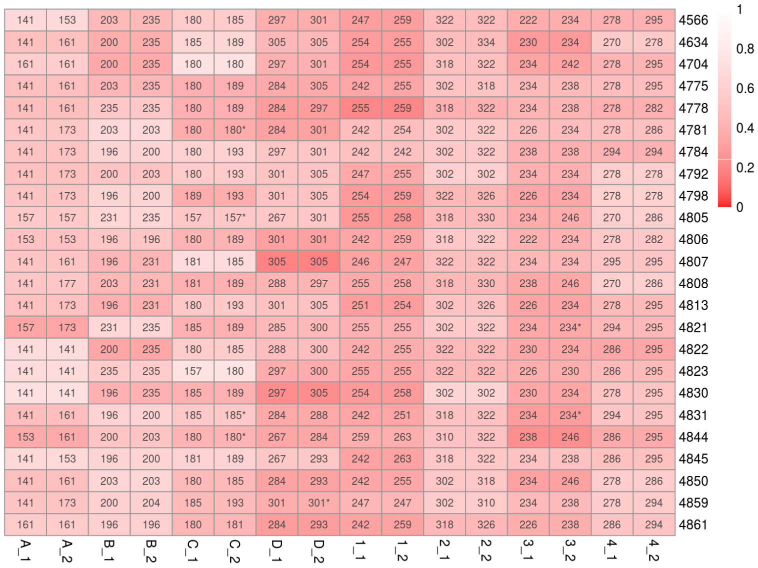

Loci with possible allelic dropout

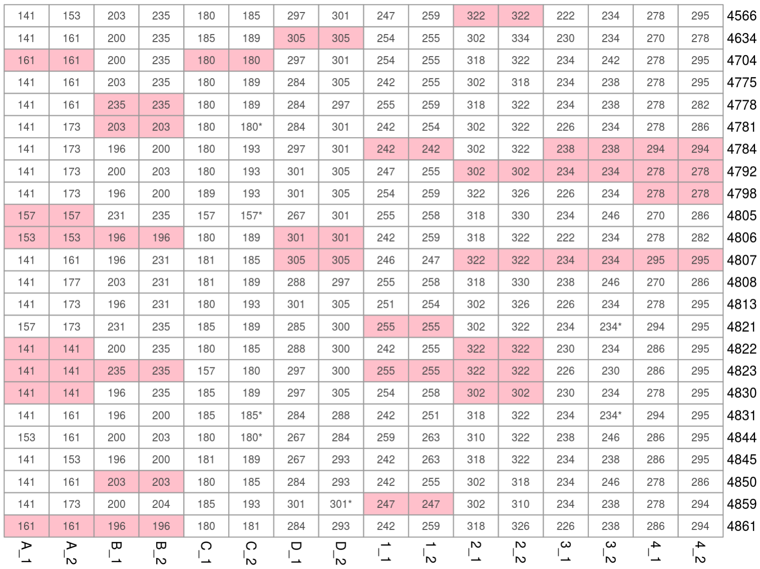

(f) Allele Alignments per Locus

Locus A

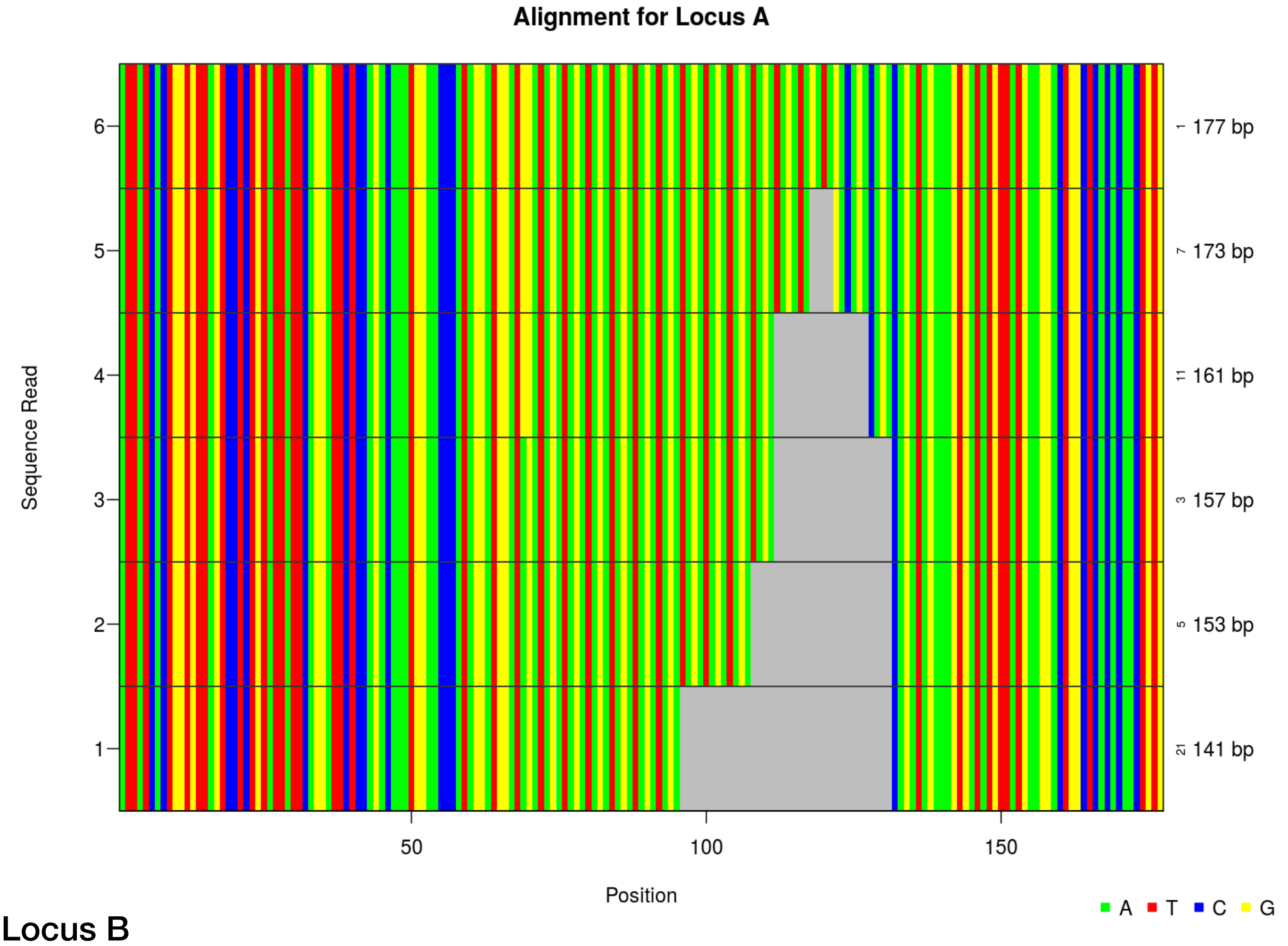

Locus B

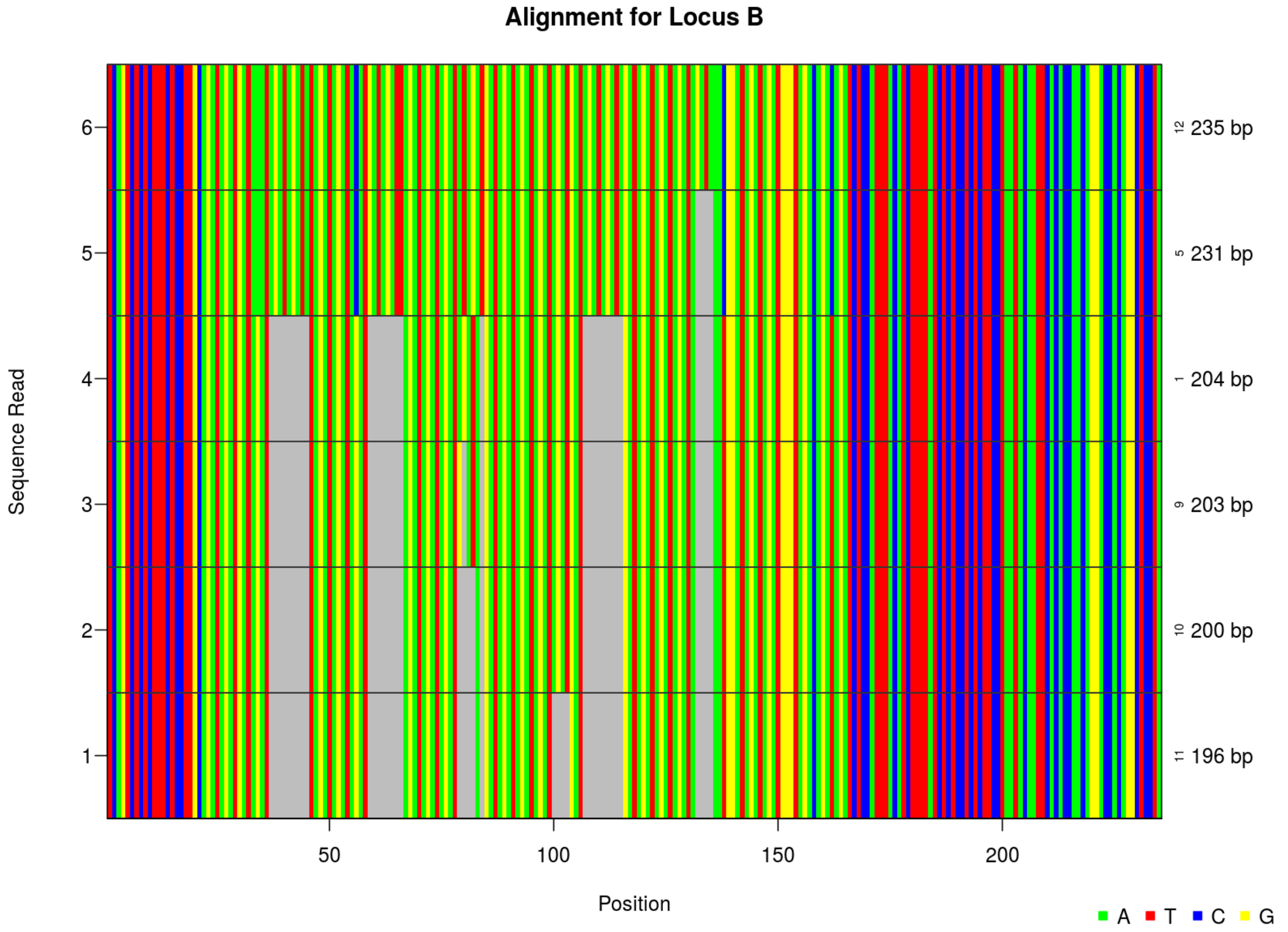

(g) Counts per Locus

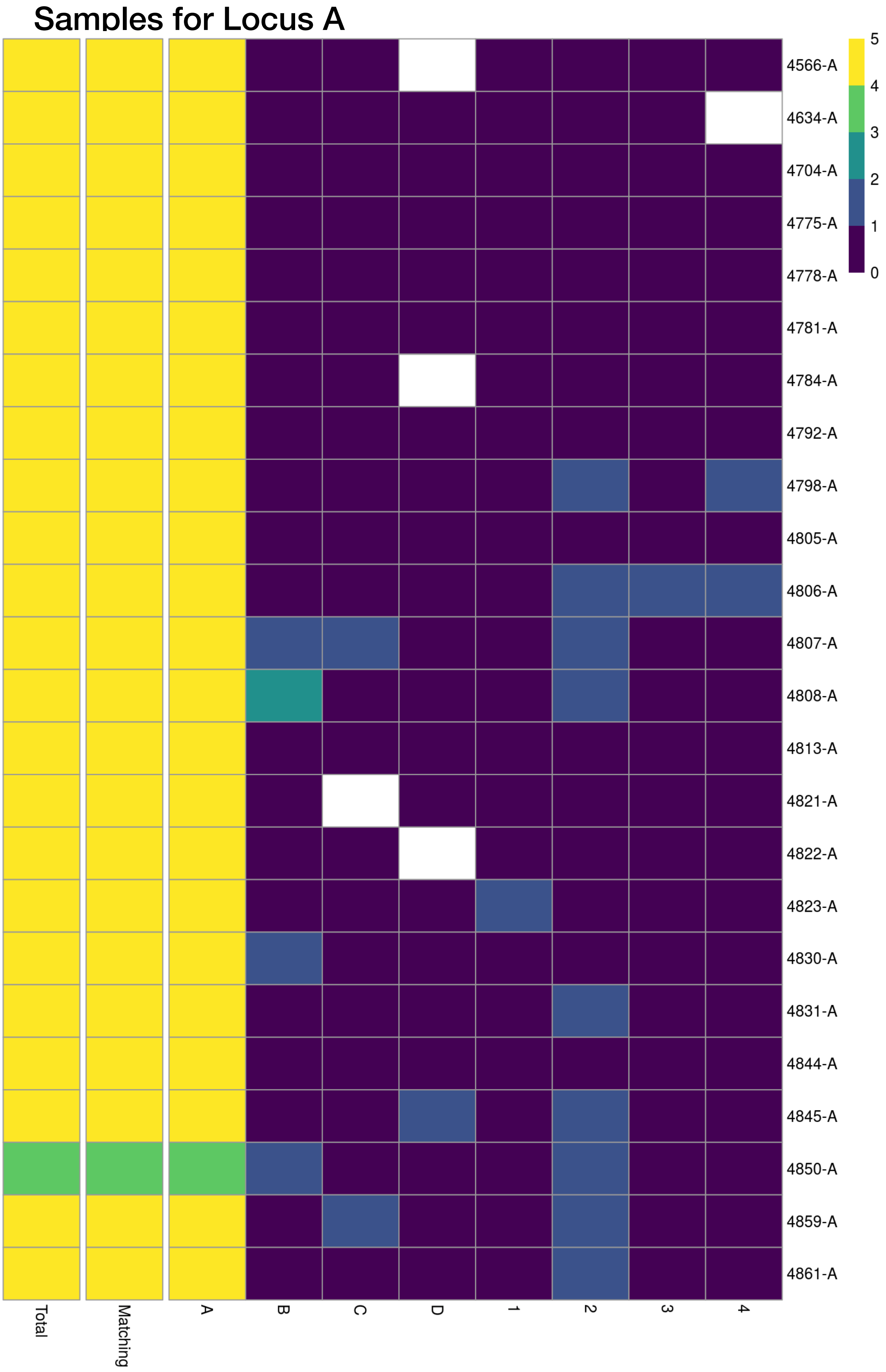

(h) Histograms

Sample 4566; Locus A, B

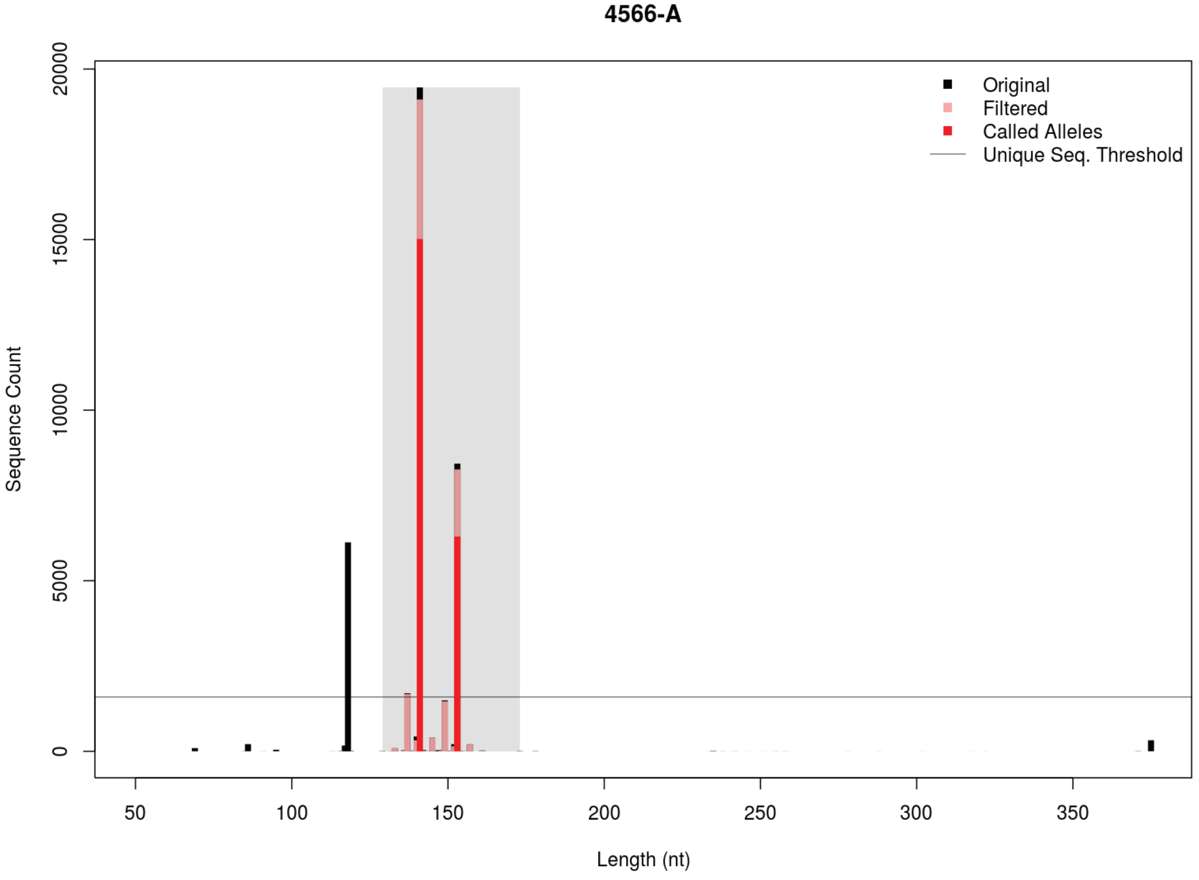

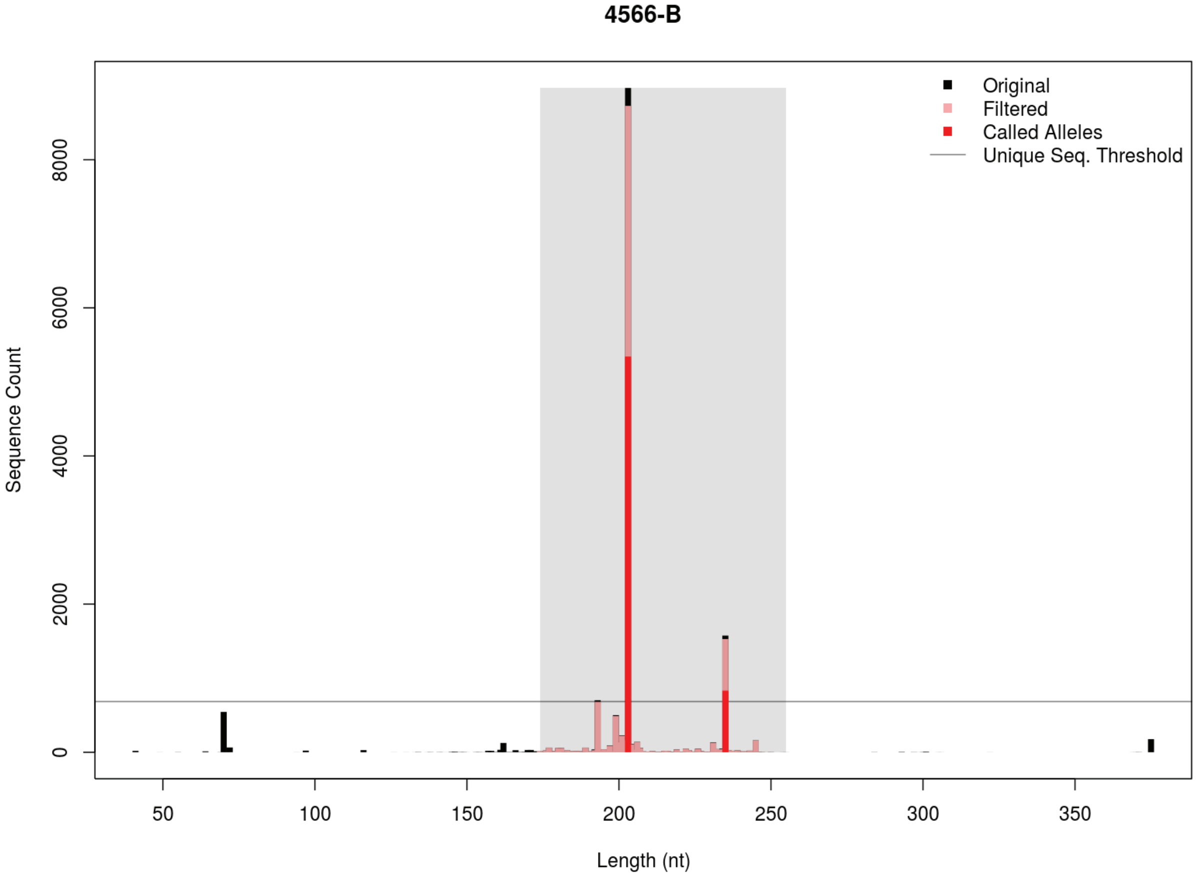

Supplement: Supplementary file 1 [file ECE3-8-7946-s001.pdf]
