# Supplementary material for: CHIIMP: An automated high‐throughput microsatellite genotyping platform reveals greater allelic diversity in wild chimpanzees
Source: Ecol Evol. 2018 Jul 16;8(16):7946–63. doi: 10.1002/ece3.4302 (PMC6145012; doi:10.1002/ece3.4302)
Supplement: Supplementary file 2 [file ECE3-8-7946-s002.docx]

**Table S1.** STR loci used for MiSeq genotyping

| Locus | Code | Forward primer | Forward primer sequence^†^ | Reverse primer | Reverse primer sequence^†^ | Size range (bp)^‡^ |
| --- | --- | --- | --- | --- | --- | --- |
| D18S536 | A | HUM05262 | 5’-ATTATCACTGGTGTTAGTCCTCTG-3’ | HUM05263 | 5’-CACAGTTGTGTGAGCCAGTC-3’ | 127-183 |
| D4S243 | B | MGS02609 | 5’-TCAGTCTCTCTTTCTCCTTGCA-3’ | MGS02610 | 5’-TAGGAGCCTGTGGTCCTGTT-3’ | 187-235 |
| D10S676 | C | HUM05148 | 5’-GAGAACAGACCCCCAAATCT-3’ | HUM05149 | 5’-ATTTCAGTTTTACTATGTGCATGC-3’ | 154-210 |
| D9S922 | D | HUM09025 | 5’-TCAGAGGACCACTGCCTAAG-3’ | HUM09026 | 5’-CTGATGGGATTTGTGCCTAT-3’ | 260-308 |
| D2S1326 | 1 | HUM09373 | 5’-AGACAGTCAAGAATAACTGCCC-3’ | HUM09374 | 5’-CTGTGGCTCAAAAGCTGAAT-3’ | 166-234 |
| D2S1333 | 2 | HUM12880 | 5’-CTTTGTCTCCCCAGTTGCTA-3’ | HUM12881 | 5’-TCTGTCATAAACCGTCTGCA-3’ | 269-357 |
| D4S1627 | 3 | HUM05068 | 5’-AGCATTAGCATTTGTCCTGG-3’ | HUM05069 | 5’-GACTAACCTGACTCCCCCTC-3’ | 202-254 |
| D9S905 | 4 | HUM07339 | 5’-GTGGGAAAATTGGCCTAAGT-3’ | HUM07340 | 5’-CTTCTGAGCCTCACACCTGT-3’ | 257-298 |

^†^STR loci were amplified as previously described (Keele *et al.* 2009b; Rudicell *et al.* 2010), except for the addition of MiSeq adapters at the 5’ end of both forward (5’-TCGTCGGCAGCGTCAGATGTGTATAAGAGACAG-3)’ and reverse (5’-GTCTCGTGGGCTCGGAGATGTGTATAAGAGACAG-3’) primers.

^‡^All previously selected loci fell within the size range of the sequencing chemistry (Illumina v2 chemistry, 500 cycle kit) and were thus sequenced without fragmentation using only the forward reads (<400 bp).
