# Supplementary material for: CHIIMP: An automated high‐throughput microsatellite genotyping platform reveals greater allelic diversity in wild chimpanzees
Source: Ecol Evol. 2018 Jul 16;8(16):7946–63. doi: 10.1002/ece3.4302 (PMC6145012; doi:10.1002/ece3.4302)
Supplement: Supplementary file 3 [file ECE3-8-7946-s003.docx]

**Table S2.** MiSeq derived genotypes for Gombe chimpanzees at eight STR loci

| Sample code | Date  collected | Chimp ID | Com.^†^ | A_1 | A_2 | B_1 | B_2 | C_1 | C_2 | D_1 | D_2 | 1_1 | 1_2 | 2_1 | 2_2 | 3_1 | 3_2 | 4_1 | 4_2 |
| --- | --- | --- | --- | --- | --- | --- | --- | --- | --- | --- | --- | --- | --- | --- | --- | --- | --- | --- | --- |
| 168 | 6/8/02 | Yolanda | KK | 157-b | 161-a | 196-a | 200-a | 180-a | 185-b | 297-b | 301-b | 242-a | 242-a | 310-a | 326-a | 234-a | 238-a | 286-a | 294-a |
| 181 | 5/4/02 | Beethoven | KK | 141-a | 157-a | 200-a | 204-a | 189-a | 189-a | 301-a | 301-a | 242-a | 259-a | 318-a | 322-a | 234-a | 238-a | 286-a | 294-a |
| 218 | 8/3/02 | Aqua | MT | 157-a | 173-a | 203-a | 235-a | 181-a | 189-b | 293-a | 297-c | 255-a | 259-a | 310-a | 322-a | 226-a | 234-a | 286-a | 295-a |
| 240 | 8/23/02 | Haiki | KL | 141-a | 177-a | 203-a | 235-a | 181-b | 181-a | 293-a | 297-a | 231-a | 255-a | 322-a | 330-a | 230-a | 246-b | 270-a | 286-a |
| 247 | 5/28/02 | Ch-085 | KL | 141-a | 161-a | 196-a | 196-a | 185-a | 189-a | 293-a | 301-a | 242-a | 255-a | 318-a | 322-a | 222-a | 238-a | 286-a | 286-a |
| 337 | 8/7/03 | Skosha | KK | 173-a | 173-a | 196-a | 196-a | 157-a | 180-a | 293-a | 297-a | 259-a | 259-a | 318-a | 322-a | 234-a | 234-a | 295-a | 295-a |
| 646 | 8/18/04 | Goblin | KK | 161-a | 173-a | 196-a | 235-a | 189-b | 189-a | 300-a | 305-a | 246-a | 259-a | 322-a | 326-a | 234-b | 234-a | 270-a | 278-a |
| 661 | 5/11/05 | Malaika | KK | 161-a | 177-a | 203-a | 203-a | 181-a | 185-a | 293-a | 301-b | 231-a | 259-a | 310-a | 322-a | 230-a | 234-a | 270-a | 286-a |
| 667 | 12/14/04 | Patti | KK | 153-a | 173-a | 196-a | 200-a | 189-b | 189-a | 267-a | 301-a | 242-a | 263-a | 302-a | 310-a | 234-a | 246-a | 270-a | 295-a |
| 715 | 5/11/05 | Echo | KK | 141-a | 177-a | 196-a | 196-a | 185-c | 189-a | 288-a | 297-a | 247-a | 247-a | 322-a | 322-a | 222-a | 238-a | 270-a | 278-a |
| 863 | 10/13/05 | Sherehe | KK | 157-a | 173-a | 200-a | 231-a | 185-b | 189-a | 301-a | 301-a | 242-a | 259-a | 302-a | 318-a | 234-a | 238-a | 286-a | 295-a |
| 981 | 11/5/05 | Gimble | KK | 141-a | 161-a | 203-a | 235-a | 189-b | 189-a | 300-a | 301-a | 242-a | 259-a | 302-a | 318-a | 226-b | 234-a | 278-a | 286-a |
| 1164 | 2/11/07 | Candy | KK | 141-a | 153-a | 196-a | 231-a | 180-a | 189-a | 288-a | 301-a | 242-a | 247-a | 318-a | 322-a | 238-a | 238-a | 270-a | 294-a |
| 1212 | 7/22/07 | Cocoa | KK | 153-a | 157-a | 196-a | 200-a | 180-a | 180-a | 288-a | 301-a | 242-a | 254-a | 318-a | 322-a | 226-a | 238-a | 294-a | 294-a |
| 1320 | 8/9/07 | Titania | KK | 141-a | 157-a | 200-a | 231-a | 180-a | 189-a | 284-a | 300-a | 242-a | 259-a | 310-a | 326-b | 222-a | 246-a | 286-a | 286-a |
| 1393 | 4/19/08 | Gremlin | KK | 141-a | 161-a | 203-a | 235-a | 180-a | 189-a | 284-a | 305-a | 242-a | 255-a | 302-a | 318-a | 234-a | 238-a | 278-a | 295-a |
| 1532 | 9/17/08 | Patina | KL | 141-a | 161-a | 200-a | 204-a | 180-a | 189-a | 297-a | 301-a | 254-a | 259-a | 322-a | 326-b | 226-a | 234-c | 286-a | 295-a |
| 1542 | 1/19/09 | Ch-106 | KL | 141-a | 177-a | 196-a | 203-a | 181-a | 189-a | 297-c | 300-a | 235-a | 255-a | 310-a | 322-a | 230-a | 234-c | 294-a | 295-a |
| 1648 | 9/8/09 | Sheldon | KK | 141-a | 161-a | 196-a | 200-a | 185-b | 185-c | 284-a | 288-a | 242-a | 251-a | 318-a | 322-a | 234-c | 234-b | 294-a | 295-a |
| 1660 | 12/8/09 | Lucy | MT | 141-a | 153-a | 200-a | 204-a | 180-a | 180-a | 267-a | 301-a | 231-a | 242-a | 318-a | 322-a | 222-a | 234-a | 278-a | 286-a |
| 1703 | 9/27/09 | Sandi | KK | 157-a | 173-a | 231-a | 231-a | 185-b | 189-a | 284-a | 301-a | 242-a | 266-a | 302-a | 302-a | 234-b | 234-a | 295-a | 295-a |
| 1705 | 7/9/09 | Ch-109 | KL | 141-a | 161-a | 200-a | 200-a | 180-a | 189-a | 285-a | 301-a | 254-a | 255-a | 302-a | 318-a | 234-c | 238-a | 278-a | 295-a |
| 1709 | 10/9/09 | Kris | KK | 141-a | 141-a | 196-a | 231-a | 180-a | 189-a | 288-a | 301-a | 242-a | 255-a | 302-a | 322-a | 238-a | 238-a | 286-a | 294-a |
| 1720 | 10/28/09 | Darbee | MT | 157-a | 173-a | 231-a | 235-a | 185-a | 189-b | 285-a | 300-a | 255-a | 255-a | 302-a | 322-a | 234-b | 234-d | 294-a | 295-a |
| 1740 | 1/3/10 | Lutana | KL | 141-a | 177-a | 203-a | 231-a | 181-a | 189-b | 288-a | 297-a | 255-a | 258-b | 318-a | 330-a | 238-a | 246-b | 270-a | 286-a |
| 1752 | 10/7/09 | Frodo | KK | 141-a | 173-a | 200-a | 203-a | 180-a | 193-a | 301-a | 301-a | 247-a | 254-a | 302-a | 322-a | 226-a | 234-a | 278-a | 286-a |
| 1827 | 2/6/10 | Hope | KK | 141-a | 161-a | 235-a | 235-a | 189-a | 189-a | 284-a | 300-a | 247-a | 247-a | 302-a | 302-a | 234-a | 238-a | 270-a | 295-a |
| 1903 | 4/14/10 | Eva | MT | 141-a | 153-a | 196-a | 235-a | 189-b | 189-b | 297-a | 301-a | 258-a | 266-a | 302-a | 322-a | 234-b | 234-a | 295-a | 295-a |
| 2142 | 10/2/10 | Baroza | KK | 141-a | 141-a | 203-a | 231-a | 185-b | 189-a | 288-a | 297-c | 255-a | 259-a | 322-a | 322-a | 222-a | 238-a | 286-a | 286-a |
| 2297 | 1/7/11 | Safi | KK | 141-a | 153-a | 196-a | 235-a | 157-a | 185-a | 297-b | 297-a | 242-a | 255-a | 318-a | 322-a | 242-a | 246-a | 270-a | 286-a |
| 2376 | 3/11/11 | Google | KK | 141-a | 161-a | 200-a | 235-a | 180-a | 189-a | 284-a | 301-a | 246-a | 259-a | 318-a | 318-a | 238-a | 238-a | 282-a | 295-a |
| 2445 | 4/21/11 | Tubi | KK | 141-a | 157-a | 203-a | 203-a | 185-a | 189-a | 267-a | 293-a | 247-a | 255-a | 302-a | 310-a | 234-a | 246-a | 294-a | 294-a |
| 2589 | 6/7/11 | Londo | MT | 141-a | 161-a | 196-a | 200-a | 180-a | 189-b | 301-a | 301-a | 247-a | 247-a | 302-a | 318-a | 234-a | 238-b | 295-a | 295-a |
| 2597 | 7/20/11 | Ferdinand | KK | 141-a | 161-a | 200-a | 235-a | 180-a | 193-a | 284-a | 301-a | 254-a | 259-a | 322-a | 322-a | 234-a | 238-a | 278-a | 286-a |
| 2641 | 8/11/11 | Ch-095 | KL | 141-a | 177-a | 200-a | 204-a | 185-c | 185-a | 301-b | 305-a | 247-a | 259-a | 310-a | 330-a | 238-a | 246-b | 282-a | 294-a |
| 2665 | 9/1/11 | Maybee | MT | 157-a | 173-a | 231-a | 235-a | 185-a | 189-a | 300-a | 300-a | 242-a | 255-a | 322-a | 322-a | 230-a | 234-d | 295-a | 295-a |
| 2673 | 9/7/11 | Apollo | KK | 141-a | 141-a | 196-a | 235-a | 157-a | 189-a | 297-a | 297-a | 255-a | 259-a | 322-a | 322-a | 234-a | 242-a | 270-a | 294-a |
| 2736 | 10/7/11 | Mambo | KK | 161-a | 161-a | 203-a | 235-a | 185-a | 189-a | 301-b | 301-a | 242-a | 259-a | 302-a | 310-a | 234-a | 234-a | 270-a | 286-a |
| 2906 | 4/9/12 | Familia | KK | 141-a | 161-a | 200-a | 200-a | 185-c | 189-a | 284-a | 301-a | 242-a | 259-a | 322-a | 322-a | 234-b | 234-b | 278-a | 294-a |
| 2935 | 4/21/12 | Bima | MT | 141-a | 153-a | 200-a | 203-a | 185-b | 185-b | 285-a | 297-c | 247-b | 259-a | 310-a | 322-a | 234-b | 234-a | 286-a | 295-a |
| 3001 | 6/8/12 | Aris | MT | 173-a | 173-a | 200-a | 235-a | 189-b | 189-a | 293-a | 301-a | 242-a | 250-a | 302-a | 322-a | 226-a | 230-a | 286-a | 295-a |
| 3011 | 6/21/12 | Titan | KK | 173-a | 173-a | 200-a | 203-a | 189-a | 193-a | 267-a | 301-a | 254-a | 263-a | 302-a | 310-a | 234-a | 234-a | 270-a | 286-a |
| 3016 | 6/24/12 | Yamaha | MT | 157-b | 161-a | 200-a | 203-a | 180-a | 185-a | 297-b | 301-b | 242-a | 246-a | 318-a | 326-a | 234-a | 246-a | 294-a | 295-a |
| 3046 | 7/11/12 | Zella | KK | 141-a | 141-a | 196-a | 204-a | 180-a | 185-a | 288-a | 305-a | 247-a | 255-a | 302-a | 310-a | 238-a | 238-a | 294-a | 294-a |
| 3049 | 7/12/12 | Zinda | KK | 141-a | 161-a | 203-a | 204-a | 185-c | 189-a | 301-a | 305-a | 242-a | 259-a | 318-a | 330-a | 234-a | 246-b | 278-a | 294-a |
| 3096 | 8/12/12 | Diaz | KK | 141-a | 161-a | 204-a | 235-a | 180-a | 180-a | 300-a | 301-a | 254-a | 255-a | 322-a | 322-a | 234-a | 234-a | 278-a | 294-a |
| 3097 | 8/12/12 | Sampson | KK | 141-a | 157-a | 231-a | 235-a | 157-a | 185-b | 284-a | 297-a | 242-a | 255-a | 302-a | 322-a | 234-b | 242-a | 294-a | 295-a |
| 3162 | 9/11/12 | Mgani | MT | 141-a | 157-a | 235-a | 235-a | 180-a | 189-b | 297-a | 301-a | 255-a | 258-b | 302-a | 318-a | 238-a | 238-a | 286-a | 295-a |
| 3165 | 9/11/12 | Loretta | MT | 141-a | 161-a | 196-a | 203-a | 181-a | 189-b | 301-a | 301-a | 247-a | 247-a | 318-a | 322-a | 234-a | 234-a | 295-a | 295-a |
| 3171 | 9/17/12 | Glama | KK | 161-a | 173-a | 200-a | 200-a | 180-a | 185-c | 305-a | 305-a | 254-a | 255-a | 302-a | 326-a | 234-c | 234-a | 278-a | 278-a |
| 3224 | 10/15/12 | Fede | MT | 141-a | 141-a | 196-a | 235-a | 189-b | 193-a | 305-b | 305-a | 242-a | 254-a | 302-a | 326-a | 234-b | 234-a | 278-a | 278-a |
| 3250 | 10/24/12 | Kipara | KK | 153-a | 157-a | 203-a | 235-a | 185-c | 189-b | 288-a | 297-a | 255-a | 266-a | 302-a | 302-a | 230-a | 234-b | 286-a | 286-a |
| 3280 | 10/31/12 | Fudge | KK | 141-a | 161-a | 200-a | 200-a | 185-c | 189-a | 288-a | 305-a | 251-a | 254-a | 318-a | 326-a | 234-c | 234-b | 278-a | 294-a |
| 3333 | 12/5/12 | Zeus | KK | 141-a | 173-a | 200-a | 204-a | 185-c | 193-a | 301-a | 301-b | 247-a | 247-a | 302-a | 310-a | 234-a | 238-a | 278-a | 294-a |
| 3348 | 12/20/12 | Edgar | MT | 141-a | 141-a | 196-a | 235-a | 185-b | 189-b | 297-a | 305-b | 254-a | 258-a | 302-a | 302-a | 230-a | 234-b | 278-a | 295-a |
| 3380 | 1/2/13 | Forest | MT | 141-a | 141-a | 231-a | 235-a | 189-b | 189-a | 300-a | 305-a | 242-a | 247-a | 322-a | 330-a | 226-a | 234-a | 278-a | 294-a |
| 3390 | 12/18/12 | Wilkie | KK | 153-a | 161-a | 203-a | 235-a | 180-a | 185-a | 284-a | 297-b | 246-a | 259-a | 318-a | 322-a | 234-a | 246-a | 282-a | 295-a |
| 3402 | 1/15/13 | Golden | KK | 141-a | 173-a | 200-a | 203-a | 180-a | 193-a | 301-a | 305-a | 247-a | 255-a | 302-a | 302-a | 234-a | 234-a | 278-a | 278-a |
| 3452 | 5/19/13 | Sindbad | KK | 157-a | 173-a | 203-a | 231-a | 180-a | 193-a | 284-a | 301-a | 247-a | 251-a | 302-a | 302-a | 234-b | 234-a | 278-a | 295-a |
| 3453 | 6/7/13 | Rudi | MT | 161-a | 173-a | 231-a | 235-a | 185-a | 189-a | 301-a | 305-b | 242-a | 246-a | 302-a | 322-a | 230-a | 234-a | 278-a | 295-a |
| 3505 | 4/20/13 | Nasa | KK | 157-b | 161-a | 196-a | 196-a | 180-a | 181-a | 284-a | 293-a | 242-a | 259-a | 318-a | 326-b | 226-a | 238-a | 286-a | 294-a |
| 3524 | 6/15/13 | Apple | MT | 161-a | 173-a | 200-a | 203-a | 180-a | 189-b | 293-a | 297-a | 255-a | 259-a | 302-a | 322-a | 234-c | 238-a | 282-a | 295-a |
| 3533 | 6/25/13 | Lutata | MT | 141-a | 141-a | 204-a | 235-a | 180-a | 189-b | 297-a | 301-a | 231-a | 258-a | 302-a | 318-a | 222-a | 230-a | 278-a | 286-a |
| 3705 | 2/4/14 | Tarzan | KK | 141-a | 173-a | 200-a | 200-a | 180-a | 189-b | 267-a | 301-a | 242-a | 254-a | 302-a | 322-a | 226-a | 246-a | 278-a | 295-a |
| 3731 | 3/9/14 | Freud | KK | 141-a | 157-a | 196-a | 200-a | 180-a | 193-a | 300-a | 301-a | 247-b | 254-a | 302-a | 322-a | 226-a | 234-a | 278-a | 294-a |
| 3746 | 6/17/14 | Gossman | KK | 141-a | 141-a | 196-a | 203-a | 185-b | 189-a | 288-a | 301-a | 242-a | 242-a | 302-a | 322-a | 234-b | 234-a | 278-a | 286-a |
| 3785 | 7/8/14 | Nuru | KK | 141-a | 141-a | 204-a | 235-a | 189-b | 189-a | 288-a | 301-a | 242-a | 259-a | 322-a | 322-a | 226-a | 234-d | 286-a | 286-a |
| 3806 | 7/16/14 | Chema | KK | 157-a | 157-a | 231-a | 235-a | 157-a | 189-a | 267-a | 301-a | 255-a | 258-a | 318-a | 330-a | 234-a | 246-b | 270-a | 286-a |
| 3807 | 7/16/14 | Duke | KK | 141-a | 173-a | 200-a | 231-b | 189-a | 193-a | 300-a | 301-a | 242-a | 254-a | 310-a | 322-a | 234-a | 234-a | 270-a | 294-a |
| 3816 | 2/20/14 | Fifti | KK | 161-a | 173-a | 196-a | 235-a | 185-a | 189-a | 284-a | 305-a | 246-a | 259-a | 318-a | 322-a | 226-a | 234-a | 278-a | 295-a |
| 3824 | 7/24/14 | Pax | KK | 141-a | 161-a | 235-a | 235-a | 180-a | 189-a | 284-a | 297-c | 242-a | 254-a | 302-a | 326-b | 234-a | 234-a | 278-a | 286-a |
| 3836 | 8/3/14 | Gaia | KK | 141-a | 161-a | 235-a | 235-a | 180-a | 189-a | 284-a | 297-b | 255-a | 259-a | 318-a | 322-a | 234-a | 238-a | 278-a | 282-a |
| 3848 | 8/7/14 | Schweini | KK | 153-a | 161-a | 196-a | 235-a | 185-b | 185-a | 284-a | 297-b | 242-a | 246-a | 318-a | 318-a | 234-a | 246-a | 282-a | 286-a |
| 3859 | 8/11/14 | Falida | MT | 141-a | 141-a | 196-a | 204-a | 189-b | 193-a | 297-a | 305-b | 242-a | 258-a | 302-a | 326-a | 234-b | 234-a | 278-a | 278-a |
| 3861 | 8/11/14 | Aphro | MT | 141-a | 173-a | 200-a | 235-a | 189-b | 189-b | 285-a | 293-a | 250-a | 255-a | 302-a | 322-a | 226-a | 234-c | 286-a | 295-a |
| 3874 | 8/16/14 | Dilly | KK | 141-a | 161-a | 204-a | 231-b | 180-a | 189-a | 300-a | 301-a | 242-a | 255-a | 322-a | 322-a | 234-a | 238-a | 286-a | 294-a |
| 3879 | 8/17/14 | Ipo | KK | 153-a | 173-a | 196-a | 235-a | 180-a | 189-a | 297-b | 301-a | 242-a | 259-a | 318-a | 322-a | 222-a | 234-a | 278-a | 282-a |
| 3884 | 8/19/14 | Eliza | KK | 141-a | 141-a | 235-a | 235-a | 157-a | 180-a | 297-a | 300-a | 255-a | 255-a | 322-a | 322-a | 226-a | 230-a | 286-a | 295-a |
| 3885 | 8/20/14 | Sparrow | KK | 157-a | 161-a | 196-a | 231-a | 180-a | 185-b | 284-a | 301-a | 242-a | 251-a | 302-a | 318-a | 234-b | 246-a | 286-a | 295-a |
| 3903 | 8/27/14 | Jiffy | KK | 141-a | 161-a | 231-a | 235-a | 189-b | 189-a | 284-a | 301-a | 247-a | 258-a | 302-a | 302-a | 234-a | 238-a | 295-a | 295-a |
| 3908 | 8/27/14 | Flossi | MT | 141-a | 161-a | 204-a | 235-a | 189-b | 193-a | 297-a | 305-a | 242-a | 259-a | 322-a | 326-a | 226-a | 234-a | 278-a | 278-a |
| 3953 | 9/29/14 | Pamera | KL | 141-a | 161-a | 196-a | 200-a | 189-a | 189-a | 297-a | 301-a | 254-a | 255-a | 322-a | 322-a | 234-c | 238-a | 286-a | 295-a |
| 3955 | 9/29/14 | Porosa | KL | 161-a | 173-a | 231-a | 231-a | 180-a | 180-a | 285-a | 293-a | 259-a | 266-b | 310-a | 322-a | 226-a | 238-a | 282-a | 294-a |
| 3958 | 10/6/14 | Vanilla | KK | 141-a | 157-a | 203-a | 203-a | 180-a | 185-b | 297-c | 297-a | 242-a | 259-a | 310-a | 322-a | 234-a | 242-a | 278-a | 294-a |
| 3961 | 10/11/14 | Losa | MT | 141-a | 161-a | 196-a | 231-a | 181-a | 185-a | 301-a | 305-b | 246-a | 247-a | 322-a | 322-a | 234-a | 234-a | 295-a | 295-a |
| 3966 | 10/22/14 | Gimli | KK | 141-a | 161-a | 203-a | 203-a | 180-a | 185-a | 284-a | 293-a | 242-a | 255-a | 302-a | 318-a | 234-a | 246-a | 278-a | 286-a |
| 3973 | 11/1/14 | Baseke | KK | 153-a | 173-a | 203-a | 203-a | 185-b | 193-a | 285-a | 301-a | 254-a | 259-a | 302-a | 322-a | 222-a | 234-a | 270-a | 294-a |
| 3974 | 11/1/14 | Bahati | KK | 141-a | 153-a | 203-a | 203-a | 185-b | 185-b | 285-a | 297-c | 247-b | 259-a | 322-a | 322-a | 222-a | 234-b | 286-a | 294-a |
| 3978 | 11/5/14 | Imani | KK | 141-a | 173-a | 196-a | 196-a | 189-a | 189-a | 288-a | 301-a | 242-a | 247-a | 318-a | 322-a | 222-a | 226-a | 278-a | 286-a |
| 3993 | 12/18/14 | Faustino | KK | 141-a | 161-a | 200-a | 235-a | 180-a | 180-a | 284-a | 301-a | 246-a | 254-a | 318-a | 322-a | 234-a | 238-a | 278-a | 295-a |
| 3996 | 12/20/14 | Eowyn | KK | 161-a | 177-a | 196-a | 196-a | 189-a | 189-a | 288-a | 301-a | 242-a | 247-a | 318-a | 322-a | 222-a | 238-a | 278-a | 286-a |
| 4000 | 12/27/14 | Flirt | MT | 141-a | 173-a | 196-a | 200-a | 180-a | 193-a | 297-a | 301-a | 242-a | 242-a | 302-a | 322-a | 238-a | 238-a | 294-a | 294-a |
| 4001 | 12/28/14 | Wema | MT | 141-a | 157-a | 231-a | 235-a | 189-b | 189-a | 267-a | 293-a | 254-a | 255-a | 302-a | 310-a | 226-a | 234-a | 270-a | 295-a |
| 4004 | 12/9/14 | Tanga | KK | 161-a | 173-a | 196-a | 200-a | 189-b | 189-a | 301-a | 305-a | 242-a | 259-a | 302-a | 326-a | 234-b | 246-a | 270-a | 278-a |
| 4063 | 1/21/15 | Pairotti | KL | 141-a | 173-a | 196-a | 231-a | 157-a | 180-a | 293-a | 293-a | 259-a | 266-b | 302-a | 310-a | 234-a | 238-a | 294-a | 295-a |
| 4109 | 7/12/15 | Nyota | KK | 141-a | 161-a | 200-a | 235-a | 185-c | 189-b | 288-a | 301-a | 242-a | 254-a | 322-a | 326-a | 234-c | 234-d | 286-a | 294-a |
| 4113 | 6/6/15 | Samwise | KK | 141-a | 173-a | 203-a | 231-a | 185-b | 193-a | 301-a | 301-a | 247-a | 266-a | 302-a | 302-a | 226-a | 234-b | 286-a | 295-a |
| 4118 | 5/30/15 | Sifa | KK | 141-a | 161-a | 196-a | 196-a | 180-a | 189-a | 293-a | 301-a | 242-a | 259-a | 302-a | 310-a | 234-a | 238-a | 286-a | 295-a |
| 4152 | 7/17/15 | Fadhila | KK | 141-a | 161-a | 200-a | 235-a | 180-a | 189-a | 284-a | 301-a | 259-a | 259-a | 322-a | 322-a | 234-b | 234-a | 278-a | 282-a |
| 4180 | 8/20/15 | Otali | MT | 153-a | 161-a | 235-a | 235-a | 180-a | 185-a | 301-a | 301-a | 242-a | 247-a | 302-a | 322-a | 230-a | 238-a | 270-a | 295-a |
| 4195 | 8/23/15 | Komoa | MT | 161-a | 177-a | 235-a | 235-a | 180-a | 189-a | 305-b | 305-b | 242-a | 255-a | 302-a | 334-a | 230-a | 246-a | 278-a | 295-a |
| 4196 | 8/22/15 | Gizmo | KK | 141-a | 161-a | 200-a | 235-a | 180-a | 180-a | 284-a | 301-a | 242-a | 254-a | 302-a | 322-a | 234-a | 238-a | 278-a | 278-a |
| 4204 | 8/23/15 | Kocha | MT | 141-a | 161-a | 200-a | 235-a | 180-a | 180-a | 301-a | 305-b | 255-a | 259-a | 322-a | 334-a | 238-b | 246-a | 270-a | 278-a |
| 4205 | 8/23/15 | Misheli | MT | 141-a | 157-a | 235-a | 235-a | 189-b | 189-b | 297-a | 301-a | 254-a | 255-a | 302-a | 318-a | 230-a | 238-a | 295-a | 295-a |
| 4206 | 8/11/15 | Tabora | KK | 141-a | 173-a | 196-a | 235-a | 180-a | 189-a | 284-a | 305-a | 242-a | 254-a | 322-a | 326-a | 234-b | 234-a | 278-a | 286-a |
| 4211 | 9/2/15 | Makiwa | KL | 141-a | 177-a | 203-a | 231-a | 181-a | 189-b | 288-a | 297-a | 255-a | 258-b | 318-a | 330-a | 238-a | 246-b | 270-a | 286-a |
| 4219 | 8/25/15 | Fansi | MT | 141-a | 161-a | 200-a | 235-a | 180-a | 193-a | 267-a | 305-a | 242-a | 259-a | 322-a | 326-b | 226-a | 234-a | 278-a | 282-a |
| 4220 | 8/25/15 | Eden | MT | 141-a | 141-a | 200-a | 235-a | 180-a | 189-b | 267-a | 301-a | 255-a | 266-a | 302-a | 322-a | 234-a | 234-a | 286-a | 295-a |
| 4223 | 8/26/15 | Kati | KL | 153-a | 161-a | 200-a | 203-a | 180-a | 189-a | 267-a | 284-a | 259-a | 263-a | 310-a | 322-a | 238-a | 246-a | 286-a | 295-a |
| 4234 | 9/26/15 | Fundi | KK | 141-a | 161-a | 196-a | 200-a | 185-b | 189-a | 288-a | 305-a | 242-a | 254-a | 322-a | 322-a | 234-b | 234-b | 278-a | 294-a |
| 4239 | 9/24/15 | Fanni | KL | 141-a | 173-a | 196-a | 200-a | 189-a | 193-a | 301-a | 305-a | 254-a | 259-a | 322-a | 326-a | 226-a | 234-b | 278-a | 278-a |
| 4247 | 9/14/15 | Trezia | KK | 141-a | 141-a | 200-a | 204-a | 185-c | 185-a | 301-b | 305-a | 247-a | 259-a | 310-a | 330-a | 238-a | 246-b | 282-a | 294-a |
| 4249 | 9/22/15 | Rumumba | KK | 153-a | 161-a | 200-a | 200-a | 180-a | 180-a | 267-a | 297-a | 231-a | 259-a | 318-a | 326-b | 234-a | 238-b | 278-a | 286-a |
| 4346 | 1/28/16 | Shwali | KK | 153-a | 173-a | 196-a | 200-a | 185-b | 189-a | 301-a | 297-b | 242-a | 263-a | 310-a | 318-a | 234-a | 234-a | 286-a | 286-a |
| 4443 | 5/6/16 | Glitter | KK | 141-a | 173-a | 203-a | 203-a | 180-a | 189-a | 284-a | 301-a | 242-a | 254-a | 302-a | 322-a | 226-a | 234-a | 278-a | 286-a |
| 4448 | 5/9/16 | Siri | KK | 141-a | 157-a | 196-a | 231-a | 189-a | 189-a | 284-a | 297-a | 259-a | 266-a | 302-a | 322-a | 234-a | 234-a | 270-a | 295-a |
| 4475 | 5/18/16 | Flower | KL | 141-a | 173-a | 235-a | 235-a | 189-b | 189-a | 305-b | 305-a | 242-a | 246-a | 322-a | 322-a | 230-a | 234-a | 278-a | 278-a |
| 4515 | 7/7/16 | Bibi | MT | 141-a | 153-a | 203-a | 235-a | 185-b | 189-b | 285-a | 297-a | 247-b | 254-a | 302-a | 322-a | 230-a | 234-a | 286-a | 295-a |
| 4517 | 7/17/16 | Lamba | MT | 153-a | 161-a | 200-a | 203-a | 180-a | 180-a | 267-a | 267-a | 231-a | 255-a | 318-a | 322-a | 222-a | 234-a | 278-a | 286-a |
| 4519 | 7/21/16 | Konyagi | MT | 161-a | 177-a | 200-a | 235-a | 180-a | 189-a | 305-b | 305-b | 255-a | 259-a | 310-b | 334-a | 230-b | 246-a | 270-a | 295-a |
| 4522 | 7/23/16 | Tom | KK | 141-a | 161-a | 196-a | 231-a | 189-a | 189-a | 288-a | 301-a | 255-a | 259-a | 302-a | 322-a | 238-a | 246-a | 270-a | 294-a |
| 4528 | 7/28/16 | Keaton | KL | 153-a | 161-a | 203-a | 203-a | 185-a | 189-b | 284-a | 297-a | 259-a | 266-a | 302-a | 322-a | 230-a | 246-a | 286-a | 295-a |
| 4534 | 7/19/16 | Kazi | KL | 141-a | 153-a | 196-a | 200-a | 181-a | 189-a | 267-a | 293-a | 242-a | 263-a | 318-a | 322-a | 234-a | 238-a | 286-a | 295-a |

^†^Gombe community: KK – Kasekela; MT – Mitumba; KL - Kalande
