# Supplementary material for: CHIIMP: An automated high‐throughput microsatellite genotyping platform reveals greater allelic diversity in wild chimpanzees
Source: Ecol Evol. 2018 Jul 16;8(16):7946–63. doi: 10.1002/ece3.4302 (PMC6145012; doi:10.1002/ece3.4302)
Supplement: Supplementary file 4 [file ECE3-8-7946-s004.docx]

**Table S3.** MiSeq genotyping of GME chimpanzees using singleplex and multiplex locus amplification

| Sample (Individual)  ( | Method | A-1 | A-2 | B-1 | B-2 | C-1 | C-2 | D-1 | D-2 | 1-1 | 1-2 | 2-1 | 2-2 | 3-1 | 3-2 | 4-1 | 4-2 |
| --- | --- | --- | --- | --- | --- | --- | --- | --- | --- | --- | --- | --- | --- | --- | --- | --- | --- |
| TZ037 | CE - historical^†^ | 139 | 159 | 230 | 234 | 175 | 183 | 286 | 298 | 231 | 259 | 321 | 321 | 221 | 221 | 281 | 293 |
| (Ch-44) | Singleplex^‡^ | 141 | 161 |  |  |  |  | 284 | 284 | 231 | 259 |  |  | 222 | 222 | 282 | 295 |
|  | One-step multiplex^§^ | 141 | 161 |  |  | 177 | 185 | 284 | 296 | 231 | 259 |  |  | 222 | 222 |  |  |
|  | Two-step multiplex^¶^ | 141 | 141 | 181 | 181 | 177 | 185 | 284 | 284 | 231 | 231 | 322 | 322 | 222 | 222 | 282 | 294 |
| TZ060 | CE - historical | 139 | 159 | 202 | 202 | 175 | 183 | 286 | 290 | 231 | 263 | 337 | 337 | 221 | 221 | 269 | 293 |
| (Ch-58) | Singleplex | 141 | 161 | 203 | 204 | 177 | 185 | 284 | 288 | 231 | 231 | 338 | 338 | 222 | 222 | 270 | 295 |
|  | One-step multiplex | 141 | 161 | 203 | 204 | 177 | 185 | 284 | 288 | 231 | 231 |  |  | 222 | 222 |  |  |
|  | Two-step multiplex | 141 | 141 | 203 | 204 | 177 | 185 | 284 | 288 | 231 | 231 | 334 | 338 | 222 | 222 | 270 | 295 |
| TZ096 | CE - historical | 139 | 175 | 202 | 230 | 183 | 183 | 286 | 290 | 251 | 255 | 317 | 321 | 221 | 233 | 281 | 293 |
| (Ch-19) | Singleplex | 141 | 141 | 203 | 231 | 185 | 185 | 285 | 288 | 251 | 255 | 318 | 322 | 222 | 234 | 282 | 295 |
|  | One-step multiplex | 141 | 177 | 203 | 203 | 185 | 185 | 285 | 288 | 251 | 255 |  |  | 222 | 234 |  |  |
|  | Two-step multiplex | 141 | 141 | 203 | 203 | 185 | 185 |  |  | 251 | 255 | 318 | 322 | 222 | 234 | 282 | 295 |
| TZ220 | CE - historical | 171 | 175 | 194 | 202 | 175 | 175 | 286 | 290 | 231 | 259 | 317 | 317 | 221 | 221 | 293 | 293 |
| (Ch-51) | Singleplex | 173 | 177 | 196 | 203 | 177 | 177 | 285 | 288 | 231 | 258 |  |  | 222 | 222 | 295 | 295 |
|  | One-step multiplex | 173 | 177 | 196 | 203 | 177 | 177 | 285 | 288 | 231 | 258 |  |  | 222 | 222 |  |  |
|  | Two-step multiplex | 169 | 173 | 196 | 203 | 177 | 177 | 288 | 288 | 231 | 231 | 318 | 318 | 222 | 222 | 295 | 295 |
| TZ254 | CE - historical | 159 | 159 | 202 | 234 | 183 | 191 | 298 | 302 | 251 | 255 | 329 | 337 | 221 | 221 | 293 | 293 |
| (Ch-26) | Singleplex | 161 | 161 | 203 | 235 | 185 | 193 |  |  | 251 | 255 |  |  | 222 | 222 |  |  |
|  | One-step multiplex | 161 | 161 |  |  | 185 | 193 | 297 | 301 | 251 | 255 |  |  | 222 | 222 |  |  |
|  | Two-step multiplex | 161 | 161 | 203 | 203 | 185 | 193 |  |  | 251 | 255 | 330 | 338 | 222 | 222 | 295 | 295 |
| TZ259 | CE - historical | 151 | 159 | 198 | 202 | 175 | 183 | 270 | 286 | 231 | 255 | 321 | 325 | 221 | 221 | 293 | 293 |
| (Ch-54) | Singleplex | 153 | 161 | 200 | 203 | 177 | 185 | 267 | 284 | 227 | 231 |  |  | 222 | 222 |  |  |
|  | One-step multiplex | 153 | 161 | 200 | 203 | 177 | 185 | 267 | 267 | 227 | 231 |  |  | 222 | 222 |  |  |
|  | Two-step multiplex | 153 | 153 | 200 | 203 | 177 | 185 | 267 | 267 | 227 | 231 |  |  | 222 | 222 | 295 | 295 |
| TZ260 | CE - historical | 159 | 175 | 218 | 230 | 175 | 183 | 286 | 298 | 251 | 262 | 317 | 325 | 221 | 233 | 285 | 293 |
| (Ch-4) | Singleplex | 161 | 177 | 219 | 219 | 177 | 185 | 284 | 296 | 251 | 262 |  |  | 222 | 234 |  |  |
|  | One-step multiplex | 161 | 177 |  |  | 177 | 181 | 284 | 296 | 251 | 262 |  |  | 222 | 234 |  |  |
|  | Two-step multiplex | 161 | 177 | 219 | 231 | 177 | 181 | 284 | 284 | 251 | 262 | 318 | 326 | 222 | 234 | 286 | 295 |
| TZ263 | CE - historical | 159 | 171 | 194 | 202 | 175 | 179 | 270 | 286 | 251 | 263 | 325 | 325 | 221 | 221 | 281 | 285 |
| (Ch-48) | Singleplex | 161 | 173 | 196 | 203 |  |  | 267 | 285 | 251 | 262 |  |  | 222 | 222 |  |  |
|  | One-step multiplex | 161 | 173 | 196 | 203 | 177 | 180 | 267 | 285 | 251 | 262 |  |  | 222 | 222 |  |  |
|  | Two-step multiplex | 161 | 161 | 196 | 203 | 177 | 180 | 267 | 285 | 251 | 262 |  |  | 222 | 222 | 282 | 286 |
| TZ264 | CE - historical | 159 | 171 | 230 | 230 | 183 | 183 | 290 | 298 | 251 | 255 | 309 | 337 | 221 | 221 | 269 | 269 |
| (Ch-53) | Singleplex | 161 | 173 | 231 | 231 |  |  |  |  | 251 | 255 |  |  | 222 | 222 |  |  |
|  | One-step multiplex | 161 | 161 |  |  | 185 | 185 | 288 | 297 | 251 | 255 |  |  | 222 | 222 |  |  |
|  | Two-step multiplex | 161 | 161 | 231 | 231 | 185 | 185 |  |  |  |  |  |  | 222 | 222 | 270 | 270 |
| TZ271 | CE - historical | 139 | 171 | 230 | 234 | 175 | 179 | 286 | 290 | 263 | 263 | 321 | 325 | 221 | 225 | 285 | 293 |
| (Ch-18) | Singleplex |  |  | 231 | 235 | 177 | 181 |  |  | 262 | 262 |  |  | 222 | 226 |  |  |
|  | One-step multiplex | 141 | 173 | 231 | 235 | 177 | 181 | 284 | 288 | 262 | 262 |  |  | 222 | 226 |  |  |
|  | Two-step multiplex | 141 | 173 | 231 | 235 | 177 | 181 |  |  |  |  | 322 | 326 | 222 | 226 | 286 | 295 |
| TZ320 | CE - historical | 175 | 179 | 194 | 198 | 183 | 191 | 270 | 290 | 263 | 263 | 321 | 321 | 233 | 233 | 269 | 293 |
| (Ch-67) | Singleplex | 141 | 177 | 196 | 200 |  |  | 267 | 289 | 262 | 262 |  |  | 234 | 234 | 270 | 295 |
|  | One-step multiplex | 177 | 181 | 196 | 200 | 185 | 193 | 267 | 289 | 262 | 262 |  |  | 234 | 234 |  |  |
|  | Two-step multiplex | 177 | 181 | 196 | 200 | 185 | 185 | 267 | 267 | 212 | 262 |  |  | 230 | 234 | 270 | 295 |
| TZ336 | CE - historical | 139 | 175 | 218 | 230 | 179 | 183 | 286 | 286 | 251 | 251 | 317 | 321 | 221 | 233 | 285 | 293 |
| (Ch-3) | Singleplex | 141 | 177 | 219 | 231 |  |  |  |  | 251 | 251 |  |  |  |  |  |  |
|  | One-step multiplex | 141 | 177 | 219 | 219 | 180 | 185 | 284 | 285 | 251 | 251 |  |  | 222 | 234 |  |  |
|  | Two-step multiplex | 141 | 141 | 219 | 231 | 180 | 180 | 284 | 284 |  |  |  |  | 222 | 234 | 286 | 295 |

^†^CE-historical: historical genotype generated previously by capillary electrophoresis (CE) analysis for samples from GME chimpanzees (Rudicell et al., 2011). All newly-derived genotypes are compared to this reference genotype.

^‡^Singleplex: genotype of the same samples generated by amplifying and MiSeq sequencing each locus individually.

^§^One-step multiplex: genotype of the same sample generated by amplifying and MiSeq sequencing two pools of four loci (one-step PCR).

^¶^Two-step multiplex: genotype of the same sample generated by using the one-step multiplex PCR product as the input for a second round PCR to then amplify each locus individually (two-step PCR). Blue cells indicate false alleles, green cells indicate stutter sequences, orange cells indicate allelic dropout, and gray cells indicate lack of amplification.
