# Supplementary material for: CHIIMP: An automated high‐throughput microsatellite genotyping platform reveals greater allelic diversity in wild chimpanzees
Source: Ecol Evol. 2018 Jul 16;8(16):7946–63. doi: 10.1002/ece3.4302 (PMC6145012; doi:10.1002/ece3.4302)
Supplement: Supplementary file 5 [file ECE3-8-7946-s005.docx]

**Supplemental Information for:**

**CHIIMP: An automated high-throughput microsatellite genotyping**

**platform reveals greater allelic diversity in wild chimpanzees**

Hannah J. Barbian, A. Jesse Connell, Alexa N. Avitto, Ronnie M. Russell, Andrew G. Smith, Madhurima S. Gundlapally, Alexander L. Shazad, Yingying Li, Frederic Bibollet-Ruche, Emily E. Wroblewski, Deus Mjungu, Elizabeth V. Lonsdorf, Fiona A. Stewart, Alexander K. Piel, Anne E. Pusey, Paul M. Sharp and Beatrice H. Hahn

**Fig. S1** The CHIIMP STR genotyping pipeline. Program outputs are shown for 24 chimpanzee fecal samples, genotyped at four polymorphic STR loci (A-D) corresponding to Table 1 and Fig. 3. (a) Summary genotype table listing sample designations for each row, STR loci for each column, and unique allele identifiers for each cell. Alleles are labeled by length, with a letter added (-a, -b, -c, etc.) to distinguish variants that differ in sequence content. Alleles that do not match previous identifiers receive a software-generated name to flag them as potentially new alleles (e.g., sample 4781, locus C, allele 2). (b) Distance matrix heatmap indicating the relative similarity of genotypes. Each cell contains a distance score, which is based on the number of allele mismatches between the respective samples (for 8 loci, the minimum is zero and the maximum is 16). Genotypes were clustered using the complete-linkage clustering algorithm in the hclust function of R (Hierarchical Clustering). More closely related genotypes are colored in darker red.  Groups of closely related genotypes (top) may reveal close relatives (or in resampled communities, specimens from the same individual) (c) Individual identification based on genotyping. Genotypes of newly collected samples (top) are compared to the genotypes of known community members, with the closest match listed below (ordered by descending distance scores). Genotypes that differ by fewer than four alleles are indicated in bold because they represent potential matches. (d) Heatmap showing the relative similarity of sample genotypes (rows) with genotypes of known individuals (columns) based on distance scores. Dark red cells indicate likely matches (Lutana and Makiwa represent the same individual). (e) Quality control tables highlighting loci where stutter sequences have been filtered, where more than two sequences pass the filter (with darker cells indicating more sequences), where a large proportion of reads is not contained in the identified alleles (light red indicates very low level of non-locus reads, indicating absence of contamination; dark red requires further scrutiny), and where homozygosity may reflect allelic dropout. (f) Alignments of allele sequences. Two representative images for locus A and B are shown.  Allele sequences are ordered by length (indicated in base pairs on the right), with the frequency with which they were found in different chimpanzees indicated on the left (the x-axis indicates the position within the alignment). Nucleotides are colored as indicated, with gaps in the alignment shown in grey. (g) Heatmap of sequence counts that match the locus-specific forward primer. A representative analysis is shown for 24 chimpanzee samples amplified at locus A. The first column shows the total number of reads. The second column shows the matching reads.  The remaining columns show the reads matching each locus (the scale bar indicates log increases; white cells indicate no reads). For singleplex samples, this identifies sequences that match other loci and thus highlights potential cross-locus contamination. For multiplexed samples, this shows the read distribution across loci. (h) Histograms depicting sequence length-frequency distributions saved as image files. Representative histograms are shown for locus A and B of one sample.  Note that peaks can be comprised of identically sized reads that differ in their sequence content and can thus contain different colors. Black highlights reads that did not match the locus length or repeat motif filter.  Pink highlights reads that appear to be locus-specific, but did not pass the PCR artifact filters (these are useful for identifying stutter sequences).  Only red reads represent true allele sequences. The horizontal line indicates the minimum read cutoff for unique sequences. Histograms from each sample and locus are saved as separate image files.
